# RNA modifications in cancer

**DOI:** 10.1002/mco2.70042

**Published:** 2025-01-10

**Authors:** Han Wu, Shi Chen, Xiang Li, Yuyang Li, He Shi, Yiwen Qing, Bohe Shi, Yifei Tang, Zhuoyi Yan, Yang Hao, Dongxu Wang, Weiwei Liu

**Affiliations:** ^1^ Department of Oral and Maxillofacial Surgery Hospital of Stomatology Jilin University, Changchun Jilin province China; ^2^ Jilin Provincial Key Laboratory of Tooth Development and Bone Remodeling Hospital of Stomatology Jilin University, Changchun Jilin provincle China; ^3^ Laboratory Animal Center College of Animal Science Jilin University, Changchun Jilin province China

**Keywords:** cancer, immune microenvironment, immunotherapy, programmed cell death, RNA modifications

## Abstract

RNA modifications are emerging as critical cancer regulators that influence tumorigenesis and progression. Key modifications, such as N6‐methyladenosine (m^6^A) and 5‐methylcytosine (m^5^C), are implicated in various cellular processes. These modifications are regulated by proteins that write, erase, and read RNA and modulate RNA stability, splicing, translation, and degradation. Recent studies have highlighted their roles in metabolic reprogramming, signaling pathways, and cell cycle control, which are essential for tumor proliferation and survival. Despite these scientific advances, the precise mechanisms by which RNA modifications affect cancer remain inadequately understood. This review comprehensively examines the role RNA modifications play in cancer proliferation, metastasis, and programmed cell death, including apoptosis, autophagy, and ferroptosis. It explores their effects on epithelial–mesenchymal transition (EMT) and the immune microenvironment, particularly in cancer metastasis. Furthermore, RNA modifications’ potential in cancer therapies, including conventional treatments, immunotherapy, and targeted therapies, is discussed. By addressing these aspects, this review aims to bridge current research gaps and underscore the therapeutic potential of targeting RNA modifications to improve cancer treatment strategies and patient outcomes.

## Graphical abstract

This review highlights the pivotal roles of key RNA modifications (m^1^A, m^5^C, m^6^A, m^7^G, ψ, and I) in cancer progression. These modifications influence tumor proliferation, metastasis, and programmed cell death, while also affecting the immune microenvironment. Understanding their mechanisms may enhance targeted therapies and improve patient outcomes in cancer treatment.

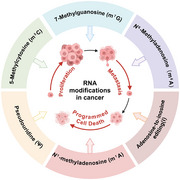

## 1 INTRODUCTION

Cancer is a significant social, public health, and economic challenge in the 21st century.^1^ In 2022, nearly 20 million new cancer cases were reported globally, resulting in 9.7 million deaths. An estimated one in five individuals will be diagnosed with cancer in their lifetimes, and approximately one in nine men and one in 12 women will succumb to the disease.^2^ Each year, cancer leaves more than a million children orphaned.^3^ It is a leading cause of premature death in many countries.^4^

The association between RNA modifications and cancer was first discovered in 1971, marking the beginning of a long journey in epigenetics research.^5^ However, limited detection methods retarded progress for many years. With advancements in RNA sequencing and quantitative fluorescence techniques, the role of RNA modifications in cancer has gained increasing attention, becoming a research focus.^6^ RNA modifications involve chemical changes to RNA nucleotides that profoundly affect RNA structure and function. To date, more than 170 RNA modifications have been identified across all RNA molecule categories.^7^ These modifications typically affect RNA splicing, stability, localization, translation, and RNA–RNA and RNA–protein interactions, thereby regulating various biological processes.^8^, ^9^, ^10^

The recent surge in research into RNA modifications highlights their potential to address the limitations of traditional cancer therapies, such as surgery, radiotherapy, chemotherapy, immunotherapy,^11^ and biotherapy. Despite these promising findings, many patients continue to face poor prognoses. Therefore, an in‐depth exploration of the molecular mechanisms driving cancer development is essential for early detection and the development of novel therapies. This review consolidates recent findings about the role of RNA modifications in cancer and cancer therapies over the past 3 years, providing insights into the current state of cancer research.

This review begins by categorizing the main types of RNA modifications, including *N* ^6^‐methyladenosine (m^6^A), 5‐methylcytosine (m^5^C), *N* ^1^‐methyladenosine (m^1^A), *N* ^7^‐methylguanosine (m^7^G), pseudouridine (Ψ), and adenosine‐to‐inosine editing. Each modification is discussed in terms of its chemical nature and biological significance. Then, the role of RNA modifications in cancer proliferation, including how they influence metabolic reprogramming, biosynthetic pathways, signaling pathway regulation, and cell cycle control, is investigated. In addition, the impact of RNA modifications on cancer metastasis is explored, focusing on mechanisms such as epithelial–mesenchymal transition (EMT) and the regulation of the immune microenvironment.

This review also addresses the involvement of RNA modifications in programmed cancer cell death, including apoptosis, autophagy, and ferroptosis, highlighting their influence on cancer cell survival strategies and the tumor immune microenvironment. Finally, the potential of RNA modifications to improve cancer therapy is discussed, particularly in the context of targeted and immunotherapies, as well as in conventional therapies and metabolism‐related treatments.

In conclusion, this review outlines the significant impact of RNA modifications on cancer proliferation, metastasis, and programmed cell death and emphasizes their potential as therapeutic targets. The types of RNA modifications are introduced first, followed by their roles in cancer proliferation, metastasis, and cell death, and then their implications for cancer therapy, to provide new insights and potential cancer treatment pathways. The majority of the referenced articles were collected from PubMed over the past 3 years to ensure its relevance and timeliness.

## 2 TYPES OF RNA MODIFICATIONS

**FIGURE 1:**
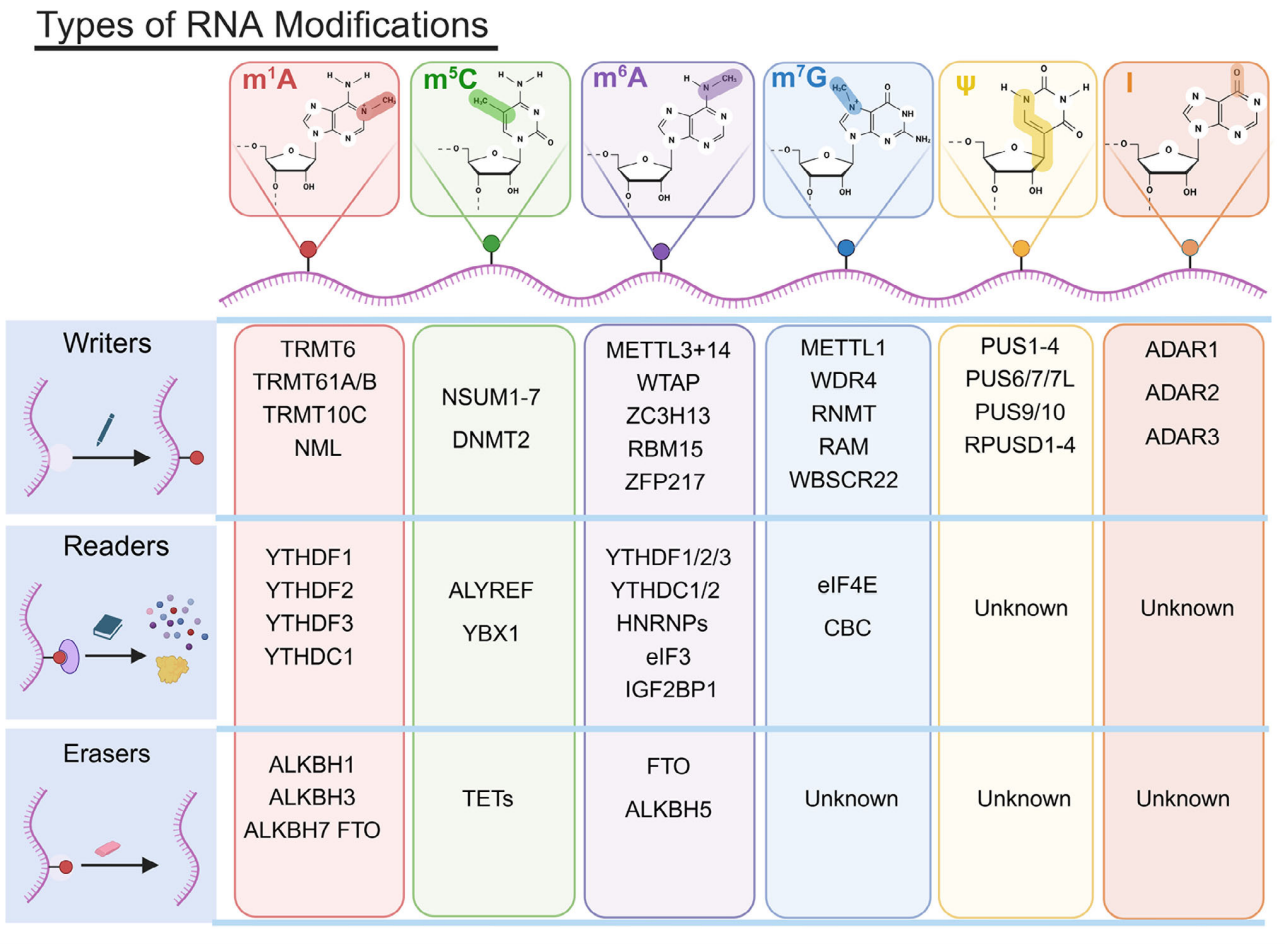
Types of RNA modifications. Figure 1 illustrates the key RNA modifications and their regulators, including writers, erasers, and readers. The modifications covered are m^1^A, m^5^C, m^6^A, m^7^G, ψ (pseudouridine), and I (adenosine‐to‐inosine editing). The specific regulators for each modification are as follows: m^1^A: writers: TRMT6, TRMT61A/B, TRMT10C, NML; readers: YTHDF1, YTHDF2, YTHDF3, YTHDC1; erasers: ALKBH1, ALKBH3, ALKBH7, FTO. m^5^C: writers: NSUN1–7, DNMT2; readers: ALYREF, YBX1; erasers: TETs. m^6^A: writers: METTL3, METTL14, WTAP, ZC3H13, RBM15, ZFP217; readers: YTHDF1/2/3, YTHDC1/2, HNRNPs, eIF3, IGF2BP1; erasers: FTO, ALKBH5. m^7^G: writers: METTL1, WDR4, RNMT, RAM, WBSCR22; readers: eIF4E, CBC; erasers: unknown. ψ (pseudouridine): writers: PUS1–4, PUS6/7/7L, PUS9/10, RPUSD1–4; readers: unknown; erasers: unknown. I (adenosine‐to‐inosine editing): writers: ADAR1, ADAR2, ADAR3; readers: unknown; erasers: unknown.

RNA modifications are a type of epigenetic modification that can cause heritable phenotypic changes without altering the nucleotide sequence of an organism's genetic material.^12^ To date, more than 170 types of RNA modifications have been identified, and all classes of RNA contain modification sites. Ribosomal RNA (rRNA) and transfer RNA (tRNA) are particularly prone to these modifications. Over 60% of RNA modifications are methylation modifications, including m^6^A,^13^ m^1^A,^14^ m^7^G, and m^5^C.^15^ Other modifications include Ψ^16^ and adenosine‐to‐inosine editing,^17^ highlighting their widespread presence and significance in complex cancer regulation^18^, ^19^ (Figure 1).

Extensive research indicates that RNA modifications are crucial in the development of various cancers, including breast,^20^ lung,^21^ and colorectal^22^ cancer, hepatocellular carcinoma (HCC),^23^ gastric,^24^ esophageal,^25^ oral,^26^ prostate,^27^ bladder,^28^ ovarian,^29^ kidney,^30^ and pancreatic cancer (PC).^31^

### 2.1 *N* ^6^‐methyladenosine

Discovered in 1974, m^6^A is the predominant RNA modification in eukaryotes.^32^ Its broad influence manifests in biological growth, development, and cancer.^33^, ^34^ m6A plays a crucial role in influencing RNA stability, transport, splicing, and translation, affecting overall RNA expression.^13^, ^35^ m^6^A modification involves the catalysis of RNA methyltransferase (the “writer”), the removal of demethylase (the “eraser”), and interaction with m^6^A‐binding protein (the “reader”). The writer includes various proteins,^36^ such as methyltransferase‐like 3 (METTL3),^20^ methyltransferase‐like 14 (METTL14),^37^ Wilms’ tumor 1 associated protein (WTAP), zinc finger CCCH‐type containing 13 (ZC3H13), RNA‐binding motif protein 15 (RBM15), vir‐like m^6^A methyltransferase associated, zinc finger protein 217 (ZFP217), and Hakai E3 ubiquitin–protein ligase (HAKAI).^38^ The primary m^6^A writer complex includes METTL3 as the catalytic subunit, METTL14 as the stabilizer, and WTAP as the regulator. This complex binds to messenger RNA (mRNA) and increases the methylation of adenosine residues.^38^, ^39^

In various cancer tissues, the *METTL3* and *METTL14* complexes play diverse roles, functioning as both oncogenes and tumor suppressors.^40^, ^41^, ^42^ m^6^A demethylases, including fat mass and obesity‐associated protein (FTO) and AlkB homolog 5 (ALKBH5), can dynamically, rapidly, and signal‐dependently influence the function of m^6^A modifications.

FTO, the first demethylase discovered, has high‐efficiency oxidative demethylation activity.^43^ FTO was initially discovered due to its role in regulating obesity and diabetes.^44^ FTO‐mediated RNA m^6^A demethylation is not specific. FTO binds multiple RNA species, including mRNA, small nuclear RNA (snRNA), and tRNA, and can demethylate internal m^6^A and cap N6,2′‐O‐dimethyladenosine (m^6^A_m_) in mRNA, internal m^6^A in U6 RNA, internal and cap m^6^A_m_ in snRNAs, and m^1^A in tRNA.^45^ The FTO gene is a multifaceted regulator, orchestrating a complex array of cellular processes.^46^ It functions by demethylating the m^6^A modification on cyclin D1 mRNA. This demethylation leads to the degradation of cyclin D1 mRNA, which impairs the progression of the G1 cell cycle phase and, ultimately, causes a decline in cancer cell proliferation.^47^ Furthermore, FTO is instrumental in regulating apoptosis, serving as a critical arbiter of cell survival and programmed cell death.^48^ The gene's influence extends to cellular motility, where it governs migration.^48^ These diverse roles underscore FTO's significance as a molecular nexus in cancer biology, with far‐reaching implications for both tumor development and potential therapeutic intervention strategies.

Meanwhile, ALKBH5 can demethylate m^6^A sequences in single‐stranded RNA (ssRNA). ALKBH5 has been employed in anti‐PD‐1 immunotherapy to regulate lactic acid levels in the tumor microenvironment (TME) and manage the accumulation of Tregs and myeloid‐derived suppressor cells (MDSCs). *ALKBH5* knockdown in a 4T1 mouse tumor model improved immunotherapy efficacy and mouse survival.^49^

The identified m^6^A readers include the YTH domain family (YTHDF)1/2/3, YTH domain‐containing protein 1/2 (YTHDC1/2), heterogeneous nuclear ribonucleoproteins A2/B1 (HNRNPA2B1), heterogeneous nuclear ribonucleoprotein C (HNRNPC), heterogeneous nuclear ribonucleoprotein G (HNRNPG), eukaryotic translation initiation factor 3 (eIF3), staphylococcal nuclease domain 1 (SND1), and insulin‐like growth factor 2 mRNA‐binding protein 1–3 (IGF2BP1–3). HNRNPA2B1 is a key regulator of proliferation, migration, and invasion in oral squamous cell carcinoma (OSCC), influencing tumor growth and metastatic potential. Its role in these processes makes it a significant therapeutic target and a potential marker for disease prognosis.^50^ YTHDF2, an m^6^A reader, is pivotal in many cellular processes, including but not limited to migration, invasion, metastasis, proliferation, apoptosis, cell cycle regulation, viability, adhesion, differentiation, and inflammation, across various neoplastic diseases.^51^, ^52^, ^53^, ^54^, ^55^ YTHDF2 exhibits differential affinities for mRNAs with m^6^A modifications; the C‐terminus specifically targets these modified mRNAs, while the N‐terminus directs the YTHDF2–mRNA complex to the cellular RNA decay checkpoint for accelerated degradation. YTHDF2's recognition of m^6^A modifications in mRNAs is essential for regulating mRNA translation and stability.^56^

### 2.2 5‐Methylcytosine

The m^5^C modification, which is catalyzed by S‐adenosylmethionine (SAM) at the carbon‐5 position of cytosine in RNA, is common in both mRNA and noncoding RNA (ncRNA). Sulfite sequencing has identified m^5^C within the coding regions of mRNA, especially near its translation initiation site.^57^ Another study discovered that m^5^C modification is also present in the untranslated region (UTR) of mRNA transcripts.^58^ m^5^C significantly influences many biological processes, such as cell proliferation, differentiation, migration, and apoptosis.^59^, ^60^

m^5^C modifications are primarily facilitated by writer proteins (methyltransferases), eraser proteins (demethylases), and reader proteins (binding proteins). m^5^C methyltransferases include NOP2/Sun RNA Methyltransferase Family (NSUN) methyltransferase and DNA methyltransferase 2 (DNMT2),^61^, ^62^, ^63^ which transfer a methyl group from adenosylmethionine to cytosine.

NSUN2 is a crucial RNA methyltransferase that introduces m^5^C into RNA. It methylates the majority of expressed tRNAs, as well as other ncRNAs and certain mRNAs.^15^, ^64^ In addition, NSUN2 functions as a direct glucose sensor, promoting tumorigenesis and resistance to immunotherapy by sustaining 3′ repair exonuclease 2 (TREX2) expression, which inactivates the cyclic GMP–AMP synthase (cGAS)/STING pathway in response to glucose activation.^65^ In colorectal cancer (CRC), NSUN2 reprograms glucose metabolism m^5^C‐dependently, increasing lactate production. Lactate accumulation in CRC cells, in turn, activates NSUN2 transcription through histone H3K18 lactylation (H3K18la) and induces NSUN2 lactylation at Lys356. This positive feedback loop of metabolic reprogramming promotes CRC progression. NSUN2 also plays a pivotal role in the progression of HCC.^67^

The principal demethylases for m^5^C belong to the ten‐eleven translocation (TET) enzyme family. Quantitative analysis via liquid chromatography–tandem mass spectrometry (LC–MS/MS/MS) indicates that TET overexpression can significantly increase the level of 5‐hydroxymethylcytosine in human embryonic kidney 293T cells.^68^, ^69^ m^5^C readers can recognize and bind to m^5^C sites on RNA to enact biological functions. Two‐dimensional liquid chromatography (2D‐LC) analysis of m^5^C‐modified RNA identified two m^5^C mRNA readers: ALY/REF export factor (ALYREF) and Y‐box‐binding protein 1 (YBX1).^70^, ^71^

Studies have shown that ALYREF can recognize and bind to m^5^C sites in mRNA, facilitating its export from the nucleus to the cytoplasm. The overexpression of ALYREF increases bladder cancer (BCa) cell proliferation by promoting pyruvate kinase M2 (PKM2)‐mediated glycolysis.^28^ Similarly, YBX1 binds to m^5^C, regulating its presence in both coding and ncRNAs and influencing rRNA maturation.^72^

### 2.3 *N^1^*‐methyladenosine

m^1^A is an ancient RNA modification found in bacteria, archaea, and eukaryotes. It involves three components: a writer, an eraser, and a reader.

The writer complex mainly comprises various methyltransferases, including the tRNA methyltransferase (TRMT)6–TRMT61A complex (for mRNA and mitochondrial tRNA),^73^ TRMT61B (for mitochondrial tRNA and rRNA),^74^ TRMT10B (for tRNA), TRMT10C (for mitochondrial tRNA and mRNA),^75^ and nucleomethylin (NML, also known as RRP8, for rRNA).^76^, ^77^ As a modification that occurs after transcription, m^1^A plays a crucial role in RNA stability by affecting base pairing.^78^

The m^1^A erasers include demethylases, such as AlkB homolog 1 (ALKBH1), AlkB homolog 3 (ALKBH3), AlkB homolog 7 (ALKBH7), and FTO, that can remove methyl groups.^79^ The enzymes FTO, ALKBH1, and ALKBH7 specifically target tRNA, while ALKBH3 acts on both tRNA and mRNA.^80^, ^81^ Although these m^1^A erasers share some functional similarities with m^6^A erasers, proteins that specifically recognize m^1^A in RNA have not yet been identified. The reader comprises various binding proteins, including YTHDF1, YTHDF2, YTHDF3, and YTHDC1.^82^

The m^1^A modification is essential in shaping the immune microenvironment and adds to the complexity of the TME. Changes in m^1^A modification patterns have been detected in several cancers, including ovarian and colon cancer, HCC, and OSCC; these changes are linked to poorer prognoses.^83^, ^84^

The m^1^A methyl group, which carries a positive charge, affects RNA base pairing, altering the structure and function of the modified RNA.^85^ During translation, m^1^A modifications affect the initiation and elongation processes by regulating tRNA, mRNA, and rRNA.^85^ Furthermore, these modifications increase the thermal stability of tRNA structures and contribute to the processing of nascent polycistronic mitochondrial RNA.^86^, ^87^

Although m^1^A is one of the most common RNA modifications in humans, its mechanisms and biological functions remain poorly understood. As m^1^A shares some regulators, such as YTHDF1–3, with m^6^A, studying m^6^A may provide insights into m^1^A. Due to its effect on RNA base pairing, m^1^A is expected to influence RNA interactions, including those involving mRNA, microRNA (miRNA), long ncRNA (lncRNA), and circular RNA (circRNA).

### 2.4 *N* ^7^‐methylguanosine

m^7^G, the methylation of guanine at the N^7^ position in RNA, occurs in roughly 0.4% of all guanosines.^88^ These m^7^G modifications are commonly located at the 5′ cap and internal sites of mRNA, as well as within rRNA, tRNA, and miRNA.^89^, ^90^ They actively affect biological and pathological processes via the metabolism of various RNA molecules.^91^

The primary enzyme responsible for this modification is METTL1, which works with the WD repeat domain 4 (WDR4) complex to enact m^7^G modifications in tRNA, miRNA, and mRNA, thereby influencing miRNA structure and biogenesis.^92^ The expression of WDR4 is closely correlated with METTL1 levels, underscoring WDR4's role as a crucial cofactor for METTL1.^93^ Reduced levels of METTL1 and WDR4 are strongly linked to neurological disorders, including brain ischemia^94^ and Alzheimer's disease.^95^ The METTL1/WDR4 complex modifies a specific subset of tRNAs with m^7^G, stabilizing these mRNAs against decay, increasing translation efficiency, reducing ribosome stalling, and increasing the expression of growth‐promoting proteins, which facilitates cancer development.^91^, ^96^, ^97^

RNA guanine‐7 methyltransferase (RNMT) and RNMT‐activating mini‐protein (RAM) are essential for effective mRNA cap methylation via m^7^G modifications.^92^ During T cell activation, stimulation of the T cell receptor induces RNMT, which coordinates the production of the mRNA, small nucleolar RNA (snoRNA), and rRNA necessary for ribosome biogenesis, improving translation capacity.^98^ Williams‐Beuren syndrome chromosome region 22 (WBSCR22) and tRNA methyltransferase 112 (TRMT112) mediate m^7^G methylation in rRNA.^99^ WBSCR22, which was originally identified as one of the 26 genes associated with Williams syndrome, contains a nuclear localization signal and a highly conserved S‐adenosyl‐l‐methionine binding motif.^100^ As a ribosome biogenesis factor, WBSCR22 has been reported in various human cancers,^101^ significantly contributing to tumor proliferation, migration, and development.^102^, ^103^

The m^7^G cap is recognized by eukaryotic translation initiation factor 4E (eIF4E) and the cap‐binding complex (CBC), influencing mRNA maturation, nuclear export, and translation.^104^

m^7^G is commonly found in mRNA, where it plays a crucial role in regulating the translation process. Its role varies across different RNA types and diseases. m^7^G promotes some cancers, such as BCa,^105^ lung cancer,^93^ liver cancer,^106^ and gliomas,^103^ but has opposite effects in teratomas^107^ and PC.^107^ Our knowledge of m^7^G regulators remains quite limited. To date, no specific demethylase has been discovered that can control m^7^G levels. The interactions between m^7^G and other posttranscriptional modifications are gaining greater attention and their underlying mechanisms invite further exploration.

### 2.5 Pseudouridine

Discovered 70 years ago, Ψ is the C5‐glycoside isomer of uridine, as well as the earliest and most abundantly modified nucleoside in RNA.^108^ Normal pyrimidine nucleosides form glycosidic bonds between the N‐1 atom of the heterocycle and the C‐1′ atom of the pentose. However, Ψ nucleosides bond the C‐5 atom of the heterocycle to the C‐1′ atom of the pentose.^109^ Ψ is found in nearly all types of RNA, both coding and noncoding, and is highly conserved across species.^110^, ^111^, ^112^

In humans, 14 Ψ writers have been identified that are responsible for catalyzing Ψ formation through either RNA‐dependent or RNA‐independent mechanisms. The RNA‐dependent process involves dyskerin Ψ synthase 1 (DKC1), which is the catalytic subunit of the H/ACA snoRNA complex that catalyzes rRNA pseudouridylation.^113^ The remaining 12 writers are the RNA‐independent single Ψ synthases (PUSs) PUS1/2/3/4/6/7/7L/9/10 and the RNA Ψ synthase domain‐containing genes 1–4 (RPUSD1–4), each with specific cellular localization and RNA targets.^114^, ^115^, ^116^, ^117^, ^118^

Currently, no specific “readers” or “erasers” for Ψ have been identified. The lack of erasers might reflect the inertness of the C−C bond between the base and ribose in Ψ compared with the C−N bond, making pseudouridylation irreversible.

When Ψ is incorporated into RNA, it increases the thermodynamic stability and spatial conformation of the RNA by increasing base stacking, improving base pairing, and rigidifying the sugar–phosphate backbone.^119^, ^120^ tRNA contains numerous pseudouridylation sites, including in the anticodon stem and loop, TΨC loop, and the D stem. These modifications contribute to tRNA's structural stability, codon–anticodon recognition, and translation efficiency and accuracy.^121^, ^122^ In rRNA, Ψ is found in critical regions such as the decoding site, mRNA channel, peptidyl transferase center, tRNA binding sites, and ribosomal subunit interface. These sites play crucial roles in ribosome assembly, function, and protein synthesis.^123^, ^124^ In mRNA, Ψ can improve precursor mRNA splicing, facilitate the conversion of nonsense codons to sense codons and improve base pairing at the ribosome decoding center, contributing to protein diversity.^111^, ^117^, ^125^ Other in vitro studies have reported that mRNA with Ψ translates more slowly and affects mRNA decoding more than unmodified mRNA.^126^

Nucleoside modifications can effectively increase mRNA stability and translation efficiency while reducing immunogenicity in vivo. The benefits conferred by Ψ make mRNA a promising tool for gene replacement and vaccination.^127^ Ψ deposition can endow modified RNA with distinct molecular properties, altering its fate or activity. In addition, as a prevalent RNA modification, Ψ plays a crucial role in various cancers.^128^, ^129^, ^130^

### 2.6 Adenosine‐to‐inosine editing

Discovered over 30 years ago, adenosine‐to‐inosine (A‐to‐I) editing was initially identified for its role in introducing a premature stop codon in mRNA, resulting in the production of apolipoprotein B.^131^, ^132^ Recently, A‐to‐I editing has been linked to tumorigenesis and cancer progression. This process is facilitated by the adenosine deaminase acting on the RNA (ADAR) family of proteins, which deaminate adenosine to produce inosine in double‐stranded RNA (dsRNA).^133^, ^134^

In humans, two catalytic forms, ADAR1 and ADAR2, mediate dsRNA‐specific A‐to‐I editing. These enzymes comprise deaminase domains and dsRNA‐binding domains (dsRBDs), functioning as both writers and editors of A‐to‐I modifications.^135^ In contrast, ADAR3 lacks editing capacity and functions as a negative regulator of ADAR1‐mediated editing. It competitively binds to dsRNA, reducing the efficiency of ADAR1 and ADAR2.^136^

In 1995, researchers reported that ADAR1 comprises two main isoforms: the interferon‐inducible ADAR1 p150 and the constitutively expressed ADAR1 p110. ADAR1 p150 is located in both the cytoplasm and the nucleus, while ADAR1 p110 primarily exists in the nucleus.^137^ In addition, ADAR1 contains a z‐DNA binding domain.^138^ Both ADAR1 and ADAR2 are ubiquitously expressed, whereas ADAR3 is predominantly found in the brain. In UTRs, A‐to‐I editing can regulate various RNA processes, including transport, translation, and degradation.^139^, ^140^, ^141^, ^142^

A‐to‐I editing facilitates the recruitment of the RNA‐binding protein human antigen R to the 3ʹ UTR of cathepsin S (CTSS) mRNA, improving the stability and translation of CTSS mRNA.^143^ In miRNAs, A‐to‐I editing can affect miRNA biogenesis and function.^144^, ^145^ Furthermore, A‐to‐I editing inhibits the formation of dsRNA structures in Alu elements, promoting canonical linear mRNA splicing and suppressing circRNA formation.^146^, ^147^ A‐to‐I editing influences lncRNAs’ secondary structure, stability, and interactions with other molecules.^148^ Adenosine deaminase acting on tRNA 2 (ADAT2) and adenosine deaminase acting on tRNA 3 (ADAT3) are closely associated with tRNA decoding capabilities.^145^

## 3 RNA MODIFICATIONS’ ROLE IN CANCER PROLIFERATION

RNA modifications play a crucial role in cancer proliferation. By regulating various metabolic pathways, biosynthetic pathways, signaling pathways, and the cell cycle, they significantly affect tumor cell growth and survival.

### 3.1 Metabolic reprogramming

**FIGURE 2:**
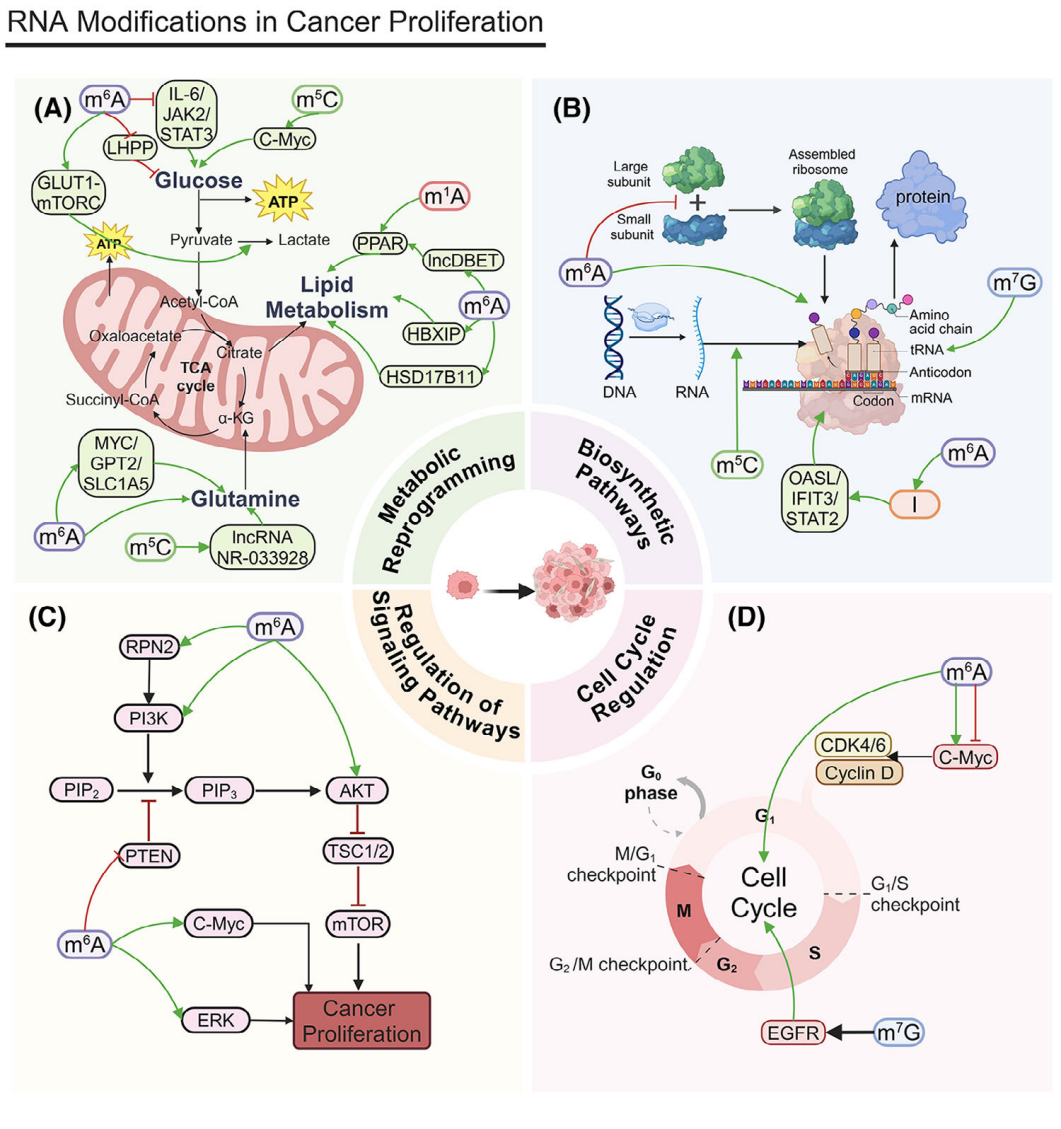
RNA modifications in cancer proliferation. (A) RNA modifications in metabolic reprogramming of cancer cells. In glycolysis, m^5^C‐modified NOP2 enhances c‐Myc expression, promoting glycolytic gene expression, while m^6^A‐modified METTL3 upregulates GLUT1 and mTORC1, enhancing glucose uptake and lactate production. METTL14 inhibits LHPP expression, promoting glycolysis. Conversely, FTO reduces APOE m^6^A modification, inhibiting the IL‐6/JAK2/STAT3 pathway. In glutaminolysis, m^5^C‐modified NSUN2 stabilizes GLS mRNA, enhancing glutaminolysis, while m^6^A‐modified METTL16 and IGF2BP2 regulate gene expression and stabilize key metabolic genes like MYC, GPT2, and SLC1A5. In lipid metabolism, m^1^A‐modified TRMT6/TRMT61A increases PPARδ translation, driving cholesterol synthesis and Hedgehog signaling, while m^6^A‐modified METTL3 and METTL14 regulate HBXIP and lncDBET, respectively, promoting lipid metabolism. FTO also regulates HSD17B11 expression, promoting lipid droplet formation. (B) RNA modifications in tumor cell biosynthetic pathways. RNMT, through m^7^G modifications on tRNAs, maintains their structural integrity and translation efficiency, thus promoting ribosome synthesis and T cell activation. m^5^C regulators like TRDMT1, NSUN1, and NSUN4 in LUAD affect RNA translation and stability, influencing tumor cell proliferation.METTL1 modulates m^6^A modifications on mRNA in the nucleus, while METTL16 interacts with eIF3a, eIF3b, and rRNA in the cytoplasm, facilitating translation initiation and promoting HCC proliferation. METTL5‐mediated m^6^A modification on 18S rRNA is crucial for 80S ribosome assembly and mRNA translation. In glioblastoma, METTL3 upregulates ADAR1 mRNA methylation, increasing its protein levels and promoting tumor growth through the METTL3/ADAR1 axis. ADAR, upregulated in breast cancer, interacts with OASL, STAT2, and IFIT3 to drive cancer progression. (C) Regulation of signaling pathways in tumor cells. FTO modulates PTEN, influencing the PI3K/AKT pathway and promoting pancreatic cancer cell proliferation and gemcitabine resistance. YTHDF1 stabilizes m^6^A‐modified mRNA, increasing RPN2 expression, which activates the PI3K/AKT/mTOR pathway, aiding bladder cancer proliferation and cisplatin resistance. High YTHDF2 expression promotes diffuse large B‐cell lymphoma (DLBCL) growth by inhibiting apoptosis and affecting ceramide metabolism and pathways like ERK and PI3K/AKT. IGF2BP2 enhances cell survival and proliferation by increasing p‐AKT and c‐myc activity. (D) Regulation of cell cycle in tumor cells. SHMT2, METTL3, FTO, ALKBH5, and IGF2BP2 regulate c‐myc mRNA stability and expression through m^6^A modifications, influencing cell cycle progression from G1 to S phase. METTL1 regulates EGFR/EFEMP1 translation, facilitating bladder cancer progression. Both FTO and ALKBH5, as m^6^A demethylases, modulate RNA methylation status, affecting gene expression related to cell cycle regulation. IGF2BP1 enhances HCC proliferation by stabilizing c‐MYC mRNA, further driving cell cycle progression from G1 to S phase.

During the metabolic reprogramming of tumor cells, the glycolytic pathway is particularly crucial (Figure 2A). Due to the Warburg effect, tumor cells preferentially use glycolysis even under aerobic conditions.^149^ This metabolic strategy not only provides the energy required for rapid proliferation but also generates precursors for biosynthesis.^150^ Glycolysis metabolites are essential for tumor cell growth and survival.^151^

Mitochondrial dysfunction and the activation of glycolysis are key characteristics of HCC. Nucleolar protein 2 (NOP2) promotes HCC progression by regulating Myc proto‐oncogene protein (c‐Myc) expression through m^5^C modification, increasing glycolysis.^152^ FTO suppresses apolipoprotein E (APOE) expression by reducing its m^6^A modification. This regulates the interleukin 6 (IL‐6)/Janus kinase (JAK2)/STAT3 signaling pathway, inhibiting glycolysis and tumor growth in papillary thyroid carcinoma (PTC) cells.^153^

In CRC, METTL3 directly induces the m^6^A–glucose transporter type 1 (GLUT1)–mechanistic target of the rapamycin complex 1 (mTORC1) axis, promoting glucose uptake, lactate production, and CRC progression. Inhibiting mTORC1 increases the anticancer effects of METTL3 silencing in CRC patients’ tissues and METTL3 transgenic mouse models.^154^ In PC, YTHDF3 interacts with lncRNA DICER1‐AS1, promoting its degradation under glucose depletion. This degradation reduces the inhibitory effect lncRNA DICER1‐AS1 imposes on glycolysis, resulting in more available energy and metabolic intermediates for rapid cancer cell proliferation.^155^ In lung adenocarcinoma (LUAD), m^6^A modification via METTL3 upregulation and ALKBH5 downregulation increases ENO1 translation, promoting glycolysis and providing energy for cancer cell proliferation. Increased ENO1 expression directly fosters tumor cell proliferation.^156^ LHPP, a histidine phosphatase, inhibits glycolysis and proliferation in gastric cancer cells by suppressing glycogen synthase kinase 3 beta (GSK3b) phosphorylation and mediating hypoxia‐inducible factor 1 alpha (HIF1A). METTL14 adds m^6^A modifications to LHPP mRNA, inhibiting its expression, which may increase GSK3b activity and reduce HIF1A activity to promote cancer cell proliferation.^157^

Glucose not only promotes tumor progression through glycolysis but also acts as a signaling molecule to regulate tumor proteins. In prostate, liver, colon, and breast cancer (BC) cells, as well as melanoma, glucose binds to the N‐terminal region of methyltransferase NSUN2, promoting its oligomerization and activation. Activated NSUN2 maintains overall m^5^C RNA methylation levels, stabilizing TREX2 and limiting dsDNA accumulation at the cell membrane and cGAS/STING activation, which promotes tumorigenesis.^65^

In addition to glycolysis, glutaminolysis is a crucial component of the metabolic reprogramming of tumor cells. Through glutaminolysis, tumor cells obtain energy and carbon sources that are essential for biosynthesis and contribute to antioxidation. Glutaminolysis is vital for tumor cell growth and survival.^158^, ^159^

NSUN2 methylates lncRNA NR‐033928, stabilizing glutaminase (GLS) mRNA and promoting glutamine metabolism reprogramming, which increases gastric cancer proliferation.^160^ Methyltransferase‐like 16 (METTL16) influences gene expression by regulating m^6^A modification, promoting genes associated with cell proliferation and supporting leukemia cell survival and proliferation.^161^ IGF2BP2 binds to m^6^A‐modified mRNA, increasing its stability and translation and thus regulating key genes involved in the glutamine metabolic pathway, such as MYC, GPT2, and solute carrier family 1 member 5 (SLC1A5), promoting acute myeloid leukemia (AML) cell proliferation and leukemia stemness/self‐renewal.^162^

The regulation of lipid metabolism also plays a vital role in the metabolic reprogramming of tumor cells. Tumor cells increase fatty acid synthesis and β‐oxidation to meet the demands of rapid proliferation. Lipid metabolism is essential not only in the construction of tumor cell membranes but also in signal transduction.^163^

The TRMT6/TRMT61A complex boosts the m^1^A methylation of specific tRNAs, increasing peroxisome proliferator‐activated receptor (PPAR) translation. This process stimulates cholesterol synthesis, activates Hedgehog signaling, and, ultimately, promotes the self‐renewal and tumorigenesis of liver cancer stem cells (CSCs).^164^ Cancer cells persistently engage in elevated glycolysis and glutaminolysis by reprogramming their internal metabolism, which drives the progression of HCC. Understanding these mechanisms can provide new insights for cancer therapies. Hepatitis B virus X‐interacting protein (HBXIP) is upregulated in liver cancer tissues and mediates the METTL3‐induced metabolic reprogramming and malignant behavior of HCC cells, accelerating tumor progression.^165^ The elevated expression of FTO correlates with poor prognoses in patients with esophageal cancer (EC). FTO promotes lipid droplet (LD) formation by regulating hydroxysteroid 17‐beta dehydrogenase 11 (HSD17B11) expression, affecting cell proliferation.^166^ In advanced BCa, m^6^A modification regulated by methyltransferase METTL14 increases the expression of lncDBET, which activates the PPAR signaling pathway and leads to increased lipid metabolism and cancer proliferation.^167^

### 3.2 Biosynthetic pathways

In tumor cell biosynthetic pathways, nucleic acid synthesis and ribosome synthesis are critical processes (Figure 2B). Tumor cells increase purine and pyrimidine synthesis to support rapid DNA and RNA replication and boost ribosome synthesis to meet high protein production demands.^168^, ^169^ These biosynthetic activities provide the necessary genetic material and proteins for rapid cell proliferation, promoting the cancer's growth and spread. This surge in nucleic acid and ribosome synthesis is closely tied to the swift proliferation of tumor cells, playing a pivotal role in their growth mechanisms.

The m^7^G methyltransferase RNMT regulates ribosome synthesis, making it essential for T cell activation. m^7^G in tRNAs maintains tRNA structural integrity by promoting stability and translation efficiency and reducing ribosome stalling, thus influencing the mRNA translatome.^98^ WBSCR22 is a ribosome biogenesis factor. The protein TRMT112 interacts with and promotes the overproduction of WBSCR22, which reduces ISG15 levels. This decrease in ISG15 expression reduces PC cells’ ability to migrate to and invade surrounding tissues.^102^

m^5^C RNA modification regulators, such as TRDMT1, NSUN1, and NSUN4, are differentially expressed in LUAD, potentially affecting RNA translation and stability and, thus, regulating tumor cell proliferation. Different m^5^C modification clusters are associated with differences in patient survival, supporting this interpretation.^170^ NOP2/NSUN1, an oncogene, is overexpressed in various cancers. By regulating rRNA processing and function, NOP2/NSUN1 may influence ribosome synthesis and promote cancer cell proliferation.^171^

In the nucleus, METTL1 regulates gene expression by modulating m^6^A modifications to mRNA. METTL16 operates in the cytoplasm, where its methyltransferase domain facilitates interactions with the eukaryotic initiation factors eIF3a and eIF3b, along with rRNA. These interactions aid in the formation of the translation initiation complex and promote mRNA translation, and they collectively promote HCC proliferation.^172^ The depletion of methyltransferase‐like 5 (METTL5)‐mediated m^6^A modification on 18S rRNA impairs 80S ribosome assembly, affecting mRNA translation and, consequently, protein synthesis related to cell proliferation.^173^ In gastric cancer, METTL3 associates with PABPC1, stabilizing the attachment of the eIF4F complex and preferentially boosting the translation of specific epigenetic factors. This mechanism enables cancer cells to more efficiently synthesize proteins related to proliferation, thereby promoting tumor growth.^174^ In glioblastoma, upregulated METTL3 methylates ADAR1 mRNA, increasing the resulting protein levels and establishing a protumor mechanism that involves METTL3, YTHDF1, and ADAR1. ADAR1 promotes cancer by binding to CDK2 mRNA independently of deaminase activity, and *ADAR1* knockdown significantly inhibits glioblastoma growth in vivo. The METTL3/ADAR1 axis establishes a connection between m^6^A modification and A‐to‐I RNA editing during posttranscriptional regulation, which reveals a novel pathway involved in cancer progression.^175^

*ADAR* is markedly upregulated in BC tissues and may drive the progression of the disease by interacting with OASL, STAT2, and IFIT3. In vitro experiments show that *ADAR* knockdown hinders BC cell proliferation, invasion, and migration, while ADAR overexpression promotes these activities.^176^ The ADAR1 enzyme performs A‐to‐I edits, preventing the sensing of endogenous dsRNA. In triple‐negative BC (TNBC) cells, *ADAR1* knockdown inhibits cell proliferation and tumorigenesis, causing strong translational suppression.

TNBC cell lines that rely on ADAR1 also demonstrate increased levels of genes activated by interferon.^177^, ^178^

### 3.3 Regulation of signaling pathways

The signaling pathway comprising phosphoinositide 3‐kinase (PI3K), protein kinase B (AKT), and mammalian target of rapamycin (mTOR) is essential for tumor cell communication, influencing cell growth, division, and maintenance (Figure 2C). The dysregulation of this pathway is strongly linked to the augmentation and persistence of numerous tumor types. Modulating this pathway can effectively influence tumor cell behavior.^179^

FTO facilitates pancreatic cancer cells’ growth and resistance to gemcitabine by modulating phosphatase and tensin homolog (PTEN) expression and affecting the PI3K/AKT signaling cascade.^180^ Meanwhile, YTHDF1 stabilizes m^6^A‐modified mRNA, boosting the expression of its downstream target, RPN2, which then activates the PI3K/AKT/mTOR pathway, thereby promoting BCa cells’ growth and resistance to cisplatin. Suppressing METTL3 and YTHDF1 expression decreases RPN2 levels, which inhibits this pathway and affects both cancer cell proliferation and chemotherapy efficacy.^181^ High YTHDF2 expression promotes diffuse large B‐cell lymphoma (DLBCL) cell proliferation and supports tumor growth by inhibiting apoptosis. YTHDF2 also influences ceramide metabolism and related pathways (e.g., extracellular signal‐regulated kinase [ERK] and PI3K/AKT), further driving tumor progression.^182^ IGF2BP2 augments cellular survival and proliferation by upregulating the activity of p‐AKT and c‐Myc. The activation of the p‐AKT pathway is intimately connected with cellular growth, metabolic processes, and survival.^183^

### 3.4 Cell cycle regulation

Cell cycle regulation is fundamental to tumor cell proliferation (Figure 2D). Cell cycle advancement is driven by cyclins and cyclin‐dependent kinases (CDKs), which together facilitate tumor cell growth. Abnormal cell cycle regulation drives the uncontrolled proliferation of tumor cells.^184^ Understanding this regulatory process is essential for elucidating tumor cell growth mechanisms.^185^

Serine hydroxymethyltransferase 2 (SHMT2) influences c‐Myc mRNA stability and expression by regulating its m^6^A modification, inhibiting c‐Myc expression through METTL3/FTO/ALKBH5/IGF2BP2 and, thereby, blocking EC cell proliferation.^186^ The oncogene c‐Myc is instrumental in advancing the cell cycle from the G1 to the S phase by upregulating genes such as Cyclin D and CDK4/6, which are essential for cellular proliferation.^187^ The tRNA m^7^G modification that is mediated by METTL1 and WDR4 is linked to unfavorable prognoses in HCC. Silencing METTL1 or WDR4 curtails HCC cell proliferation, migration, and invasion; conversely, elevated METTL1 expression facilitates HCC progression.^97^ METTL1 regulates epidermal growth factor receptor (EGFR)/EFEMP1 translation by modifying certain tRNAs, leading to BCa proliferation, migration, and invasion.^105^ FTO, an m^6^A demethylase, regulates RNA methylation status, affecting the cell cycle and proliferation‐related gene expression. The upregulation of FTO may promote uterine leiomyosarcoma cell proliferation.^188^ ALKBH5 demethylates m^6^A modifications, influencing gene expression and cell processes, including proliferation. Inhibiting ALKBH5 increases m^6^A levels, which generally decreases mRNA stability and translation efficiency, thereby affecting cell cycle regulation.^189^ This inhibition can lead to reduced cancer cell proliferation by altering mRNA dynamics.^190^ IGF2BP1 promotes HCC proliferation by regulating c‐Myc expression.^191^

In summary, RNA modifications play pivotal roles in metabolic reprogramming, biosynthetic pathways, signaling pathway regulation, and cell cycle control in tumor cells. These mechanisms collectively affect tumor cells’ rapid proliferation, driving cancer progression. A thorough investigation of RNA modification mechanisms and functions deepens our comprehension of tumor biology and offers potential targets for new cancer treatment strategies.

## 4 RNA MODIFICATIONS IN CANCER METASTASIS

The role of RNA modifications in cancer metastasis is also garnering increasing attention. These modifications influence tumor cell migration and invasion through various mechanisms. This section will consider the effects of RNA modifications in EMT, immune microenvironment regulation, signaling pathway modulation, and metabolic reprogramming.

### 4.1 Epithelial–mesenchymal transition

**FIGURE 3:**
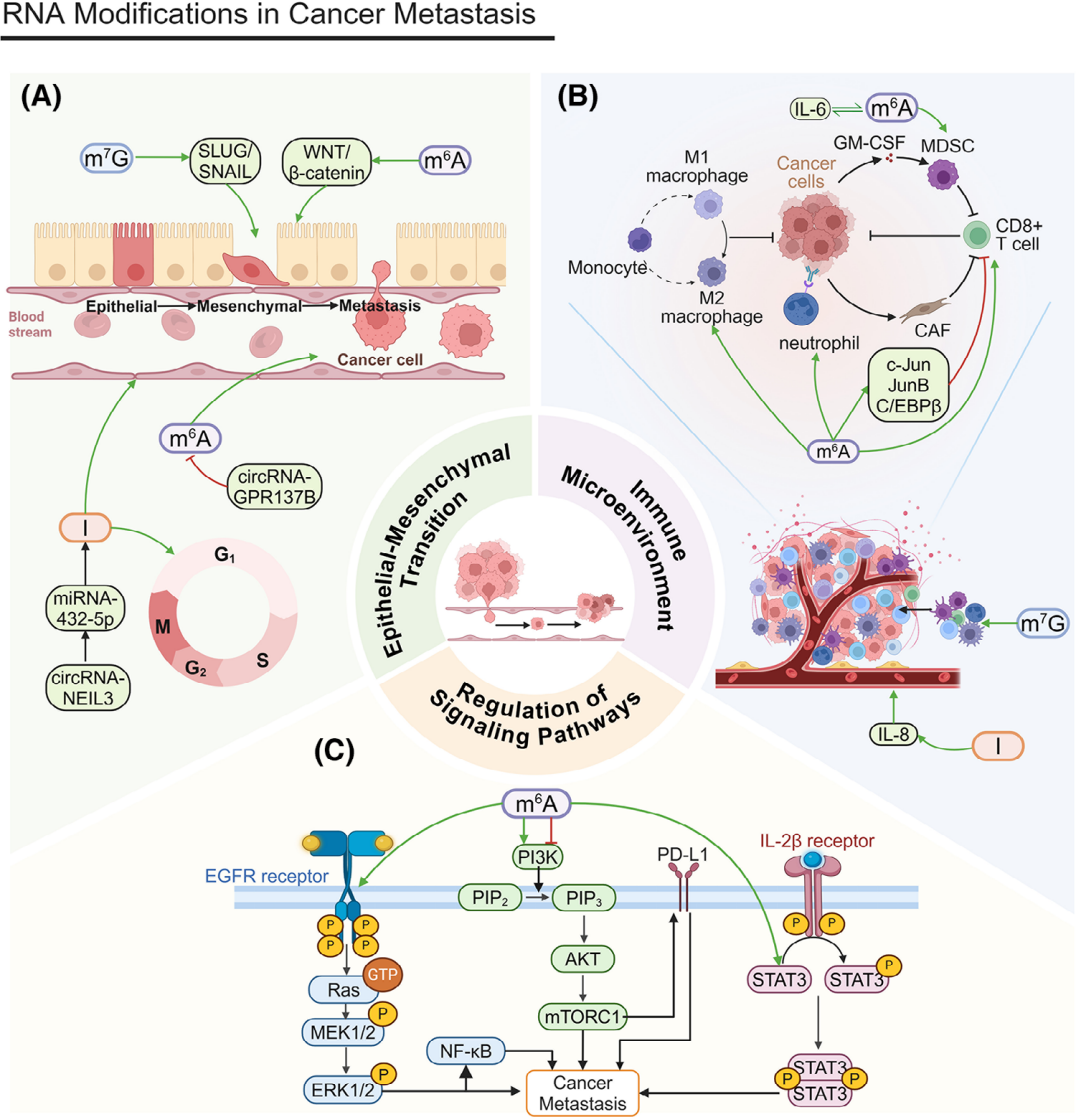
RNA modifications in cancer metastasis. (A) RNA modifications in epithelial–mesenchymal transition. YTHDF2 overexpression induces EMT, enhancing cell migration and invasion. CircNEIL3 promotes proliferation and metastasis through the circNEIL3/miR‐432‐5p/ADAR1/GLI1/cell cycle axis. METTL1 activates the WNT/β‐catenin pathway, facilitating EMT and proliferation. m^7^G modification enhances EMT by promoting SLUG/SNAIL translation, increasing invasion, especially after radiofrequency ablation. MIR100HG, regulated by m^6^A modification, stabilizes TCF7L2 mRNA, activating the Wnt/β‐catenin pathway and promoting invasion. METTL14 regulates m^6^A modification of lncRNA–NEAT1_1, with YTHDF2 accelerating its degradation to inhibit metastasis. (B) RNA modifications in regulation of the immune microenvironment. m^6^A modifications by METTL14, FTO, and METTL3 modulate immune cell functions and pathways, such as macrophage response, CD8+ T cell activity, and MDSC migration, impacting tumor immune evasion and metastasis. ALKBH5 and m^7^G modifications further regulate immune cell infiltration and the tumor–stroma interaction, enhancing cancer cell migration and invasion. A‐to‐I RNA editing of AZIN1 promotes tumor angiogenesis and metastasis by upregulating IL‐8. (C) RNA modifications in regulation of signaling pathways. m^6^A modifications by METTL14, FTO, and METTL3 affect the PI3K/AKT and MAPK/ERK pathways, modulating cell proliferation, migration, and immune evasion. METTL3 and ALKBH5 upregulate key pathway components, enhancing tumor metastasis.

EMT plays a critical role in normal embryonic development and tissue repair (Figure 3A). However, its abnormal reactivation is linked to tumor cells’ malignant traits during cancer advancement and metastasis. EMT equips tumor cells with increased migratory and invasive properties by downregulating epithelial markers, such as E‐cadherin, and upregulating mesenchymal markers, including N‐cadherin and vimentin.^192^

YTHDF2 overexpression triggers EMT in lung squamous cell carcinoma (LUSC), promoting greater cell migration and invasion. This phenomenon has been substantiated by Transwell invasion assays and wound healing assays.^193^ In pancreatic ductal adenocarcinoma (PDAC), circNEIL3 enhances proliferation and metastasis through the circNEIL3/miR‐432‐5p/ADAR1/GLI1 axis, affecting both the cell cycle and EMT processes. Its expression is modulated by ADAR1 through a negative feedback mechanism.^194^ In nasopharyngeal carcinoma (NPC), METTL1 facilitates proliferation and EMT by activating the WNT/β‐catenin signaling pathway.^195^ In HCC, m^7^G modification increases EMT by promoting the translation of SLUG and SNAIL, key EMT regulators, increasing cell invasion and metastasis, especially after radiofrequency ablation (RFA).^196^

*FTO* knockdown significantly inhibits the invasion and stemness of EC cells, while FTO overexpression promotes these traits. Invasion and stemness are crucial for cancer cell migration and metastasis, and FTO enhances EC cell migration and invasion.^166^ circGPR137B inhibits distant metastasis in HCC by upregulating FTO.^197^ MIR100HG is a positive regulator of EMT; m^6^A modification strengthens the association between MIR100HG and hnRNPA2B1, stabilizing TCF7L2 mRNA. This activation of the Wnt/β‐catenin signaling pathway promotes CRC cell invasion and metastasis.^198^ METTL14 regulates m^6^A modification of lncRNA–NEAT1_1, affecting renal cell carcinoma (RCC) cell migration. YTHDF2 selectively recognizes m^6^A modifications on NEAT1_1 and accelerates its degradation, inhibiting cell metastasis and demonstrating the critical role m^6^A modification plays in cancer metastasis.^199^

### 4.2 Regulation of the immune microenvironment

Another crucial factor in cancer metastasis is the regulation of the tumor immune microenvironment (Figure 3B). RNA modifications significantly affect tumor immune evasion and metastatic potential by altering immune cell function and infiltration. These modifications crucially mediate interactions between tumor cells and immune cells.^200^, ^201^, ^202^

The loss of METTL14 exacerbates the macrophage response to acute bacterial infections and drives CD8+ T cells toward dysfunction, impairing their ability to eliminate CRC tumors.^203^ FTO‐mediated m^6^A demethylation increases the levels of the transcription factors c‐Jun, JunB, and C/EBPβ in tumor cells, which alters glycolytic metabolism, impairs CD8+ T cell function, and inhibits tumor progression. Furthermore, m^6^A modifications alter the tumor immune microenvironment by modulating immune cell infiltration, contributing to cancer metastasis.^204^ The accumulation of MDSCs is intricately linked to tumor metastasis. METTL3 facilitates MDSC migration through the basic helix‐loop‐helix family member e41 (BHLHE41)–CXC motif chemokine ligand 1 (CXCL1)/CXCR2 axis, which may influence tumor cells’ metastatic potential.^205^ The subcellular localization of METTL3 plays a critical role in cancer metastasis. An IL‐6‐dependent positive feedback loop amplifies the function of nuclear METTL3, initiating BC metastasis. In addition, METTL3 deacetylation and nuclear translocation are associated with increased m^6^A modification levels, which may influence the expression of genes related to metastasis and, ultimately, promote it.^206^ ALKBH5 promotes cancer cell migration and invasion by modulating m^6^A modifications, a phenomenon confirmed by studies on glioma cells in vitro and in vivo. It also recruits M2 macrophages into the TME, potentially promoting tumor metastasis.^207^

m^7^G modification may increase cancer cells’ invasiveness and promote metastasis by altering the composition of immune cells within the TME. An immunosuppressive microenvironment can diminish the immune system's ability to monitor tumor cells, elevating the risk of cancer cell metastasis.^208^ Core m^7^G‐modified genes *FN1* and *ITGB1* regulate immune cell infiltration (e.g., macrophage and neutrophil upregulation) by fostering interactions between the stroma and cancer cells, driving invasion and metastasis.^209^ Although glioma metastasis is less pronounced than that of other cancers, m^7^G can still influence tumor cell invasion via the SPP1 and PTN signaling pathways, facilitating its expansion into surrounding tissues.^210^

A‐to‐I editing of AZIN1 promotes tumor angiogenesis and increases tumor cell invasion and metastasis by upregulating IL‐8.^211^ Having completed our discussion of the effects of RNA modifications on the immune microenvironment, we will next analyze their role in signaling pathway regulation.

### 4.3 Regulation of signaling pathways

The regulation of signaling pathways is essential for tumor cell migration and invasion (Figure 3C). RNA modifications affect tumor cell behavior through multiple signaling pathways, such as the PI3K/AKT pathway, the MAPK/ERK pathway, and others.

The PI3K/AKT pathway is fundamental for cell growth, proliferation, and survival. The upregulation of fibroblast growth factor 19 (FGF19) and its receptor, fibroblast growth factor receptor 4 (FGFR4), is associated with HCC cells’ invasiveness. By driving IGF2BP1 expression, FGF19/FGFR4 may foster metastasis. These proteins can promote programmed death ligand 1 (PD‐L1) upregulation via the PI3K/AKT pathway, boosting tumor cells’ immune evasion and metastatic potential.^212^ METTL14 upregulation also inhibits RCC cell migration. The PI3K/AKT pathway is integral not only to cell proliferation but also to cell migration and invasion. By inhibiting this pathway via m^6^A modification, METTL14 indirectly suppresses cancer cell migration and invasion.^213^ FTO inhibitors may also alter tumor cell invasiveness by affecting cell proliferation and survival, influencing their metastatic capacity. Furthermore, FTO inhibitors can regulate the Wnt/PI3K–AKT signaling pathway, affecting cancer metastasis.^214^ The expression of EphA2 and vascular endothelial growth factor A (VEGFA) are closely linked to tumor metastasis. Research indicates that METTL3, through an IGF2BP2/3‐dependent mechanism, inhibits the degradation of EphA2 and VEGFA mRNA by modulating the PI3K/AKT and ERK1/2 pathways, facilitating metastasis.^215^

The MAPK/ERK pathway facilitates tumor cell growth and migration by controlling genes related to the cell cycle and cell proliferation. RNA modifications are also crucial in this pathway. ALKBH5 boosts both the proliferation and metastasis of HCC cells by upregulating MAP3K8, which, in turn, activates the JNK and ERK signaling pathways that are essential for cell migration and metastasis.^216^ Deficiencies of the m^6^A methyltransferase METTL3 hinder YTHDF1‐mediated translation of Sprouty‐related EVH1 domain‐containing protein 2 (SPRED2), augmenting the activation of nuclear factor kappa B (NF‐κB) and STAT3. This augmentation promotes tumor growth and metastasis through the ERK pathway.^217^ METTL3 also plays a pivotal role in cancer metastasis through its regulation of the MAPK signaling pathway and related immune responses.^218^

In addition to these pathways, RNA modifications influence tumor cell migration and invasion through other signaling pathways.

METTL3 governs the m^6^A modification of *Frizzled‐10*, increasing its expression in liver cancer stem cells and activating the β‐catenin and yes‐associated protein 1 (YAP1) signaling pathways. This increases tumor cell self‐renewal and tumorigenicity. The upregulation of *Frizzled‐10* forms a positive feedback loop, further activating METTL3 and driving HCC metastasis and drug resistance.^219^

FTO not only promotes cell proliferation but also supports tumor metastasis by affecting cell migration and invasion. It regulates heat shock factor 1(HSF1)Heat Shock ProteinsHeat Shock Proteins expression through demethylation, altering HSP levels, which promotes the proliferation, survival, migration, and invasion of multiple myeloma cells.^220^ Carbon ion radiotherapy elevates METTL3 levels and its associated m^6^A modifications in NSCLC cells, influencing cancer cell migration and invasion. By regulating critical gene expression, m^6^A modifications can either promote or suppress cancer metastasis.^221^ The overexpression of WTAP markedly increases ovarian cancer cells’ invasive potential by modulating miR‐200 expression and affecting genes involved in cell migration and invasion.^222^ The upregulation of METTL16 in HCC induces m^6^A modifications of lncRNA–RAB11B‐AS1, decreasing its transcript stability and resulting in its downregulation. This process promotes the proliferation, migration, and invasion of HCC cells, inhibits apoptosis, and facilitates tumor growth in vivo.^223^

### 4.4 Metabolic reprogramming in cancer metastasis

Metabolic reprogramming is essential for tumor cells to adapt to their rapid proliferation and challenging environmental conditions. Because RNA modifications regulate metabolic pathways, affecting energy metabolism and biosynthesis, they can enhance migration and invasion.

*YTHDF1* increases BC cell tumorigenicity and metastasis by upregulating *PKM2*, promoting glycolysis. Inhibiting *YTHDF1* eliminates *YTHDF1*‐dependent tumor growth and metastasis.^224^ Targeting METTL3/14 increases ACLY and SCD1 protein levels, along with the production of triglycerides and cholesterol and the accumulation of LDs. This change in membrane lipid composition fosters tumor cell migration and invasion.^225^ Mitochondrial m^5^C modification is essential for cancer cell metastasis. Tumor cells that are dependent on *CD36* rely on m^5^C modifications to initiate invasion and dissemination. Cells deficient in m^5^C exhibit impaired metastasis, underscoring the significance of RNA modifications in modulating mitochondrial oxidative phosphorylation and metabolic pathways.^226^

In conclusion, RNA modifications drive numerous mechanisms underlying cancer metastasis. A comprehensive investigation of the specific functions and mechanisms of these modifications will improve our understanding of the intricate processes driving tumor metastasis and offer novel insights for future therapeutic strategies.

## 5 RNA MODIFICATIONS IN PROGRAMMED CANCER CELL DEATH

RNA modifications’ role in cancer programmed cell death is gaining increasing attention as these modifications influence tumor cell survival and death through various mechanisms. This section will detail how RNA modifications affect different types of programmed cell death through signaling pathway regulation and altering the tumor immune microenvironment.

### 5.1 Types of programmed cell death

**FIGURE 4:**
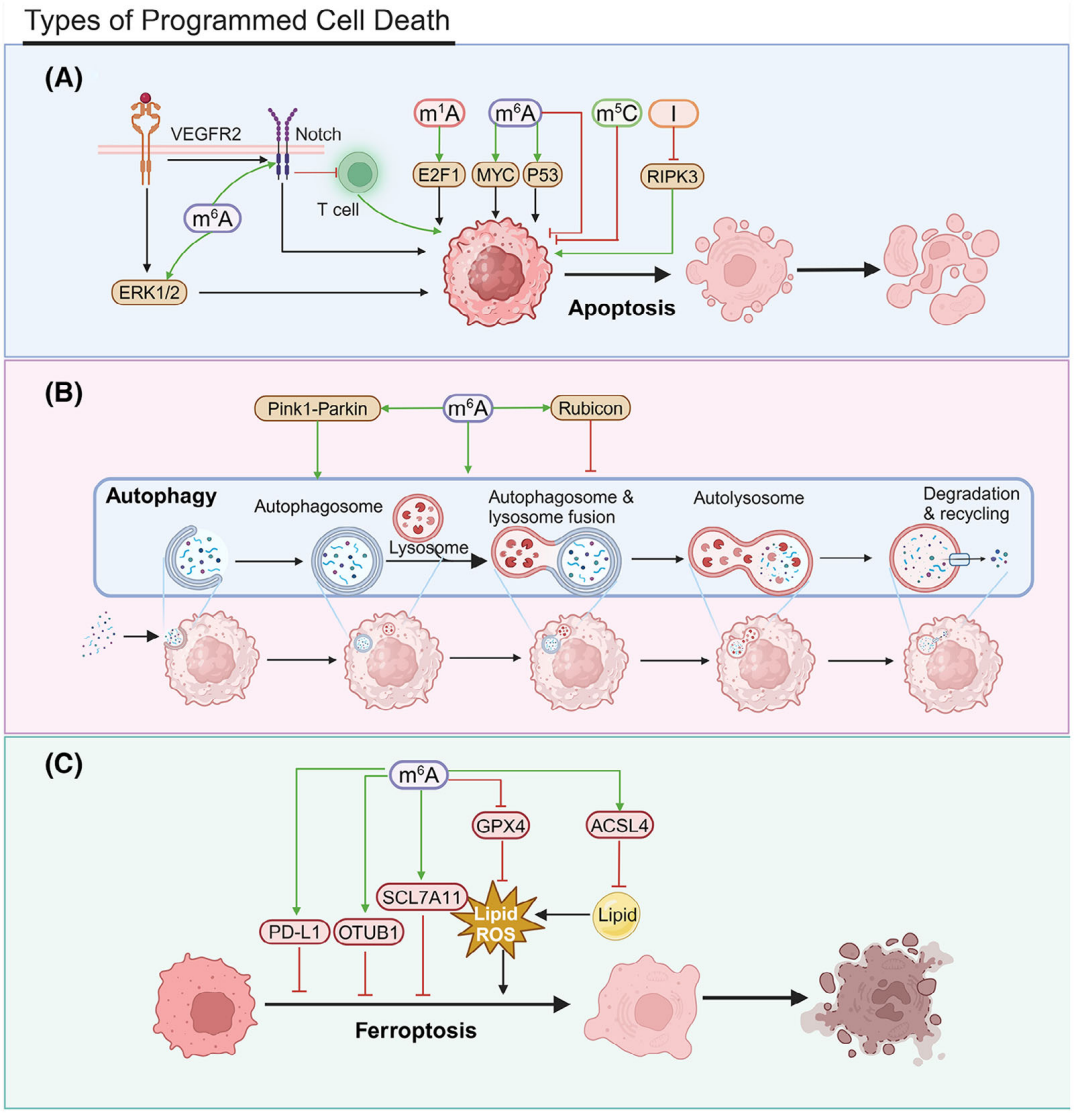
Types of programmed cell death. (A) RNA modifications in apoptosis. m^1^A affects E2F1 mRNA stability, regulating cell cycle and apoptosis. ALYREF depletion increases apoptosis rates. YTHDF1 upregulation inhibits antitumor immune responses, affecting apoptosis. FTO inhibitors enhance SOCS1 stability and activate the P53 pathway, promoting apoptosis. FTO inhibitors also regulate RARA and MYC expression, inducing apoptosis. IGF2BP2 affects mitochondrial activity and gene expression, influencing cell survival. IGF2BP1 knockout promotes apoptosis. CuB blocks IGF2BP1 recognition of m^6^A‐modified mRNA, promoting apoptosis. High FTO expression inhibits apoptosis via the PDGFRB/ERK axis. IGF2BP2 knockdown increases apoptosis through m^6^A modifications. ADAR1 inhibits necroptosis by preventing ZBP1 activation. (B) RNA modifications in autophagy. m^6^A modifications regulate autophagy‐related genes, influencing cell survival and death. METTL3 targets DCP2, regulating the Pink1–Parkin pathway and affecting mitochondrial autophagy and damage. METTL3 and YTHDF1 enhance Rubicon mRNA stability, inhibiting autophagosome–lysosome fusion and lipid droplet clearance. (C) RNA modifications in ferroptosis. FTO demethylates OTUB1 mRNA, inhibiting ferroptosis and enhancing radioresistance. FTO downregulates SLC7A11, promoting ferroptosis. METTL3 recruits YTHDF1 to enhance SLC7A11 m^6^A modification and translation, inhibiting ferroptosis. METTL3/IGF2BP1/m^6^A axis stabilizes SLC7A11 mRNA, preventing deadenylation and promoting cell proliferation. m^6^A–SLC7A11 upregulation confers ferroptosis resistance. Hypoxia‐induced lncRNA–CBSLR interacts with YTHDF2, reducing CBS mRNA stability and affecting ACSL4 protein, leading to ferroptosis resistance. CD8 T cells stimulate ACSL4 expression, promoting ferroptosis. PRMT3 inhibition‐mediated METTL14 upregulation reduces GPX4 mRNA stability, increasing lipid peroxidation and ferroptosis. YTHDF1 inhibits CD8+ T cell‐mediated ferroptosis, affecting programmed cell death.

Programmed cell death is an active cellular process that occurs under particular conditions, encompassing apoptosis, autophagy, and ferroptosis. Apoptosis is triggered by internal and external signals involving enzymes, such as caspases, and various signaling pathways (Figure 4A). Many anticancer drugs kill tumor cells by inducing apoptosis.

The m^1^A modification influences the stability of E2 promoter binding factor 1 (E2F1) mRNA, a key regulator of the cell cycle and apoptosis. By regulating E2F1 expression, m^1^A can indirectly affect programmed cell death in cancer cells.^227^ The m^5^C reader ALYREF is notably upregulated in HCC tissues and cell lines. *ALYREF* knockdown markedly suppresses the proliferation of Huh7 and HepG2 cells and elevates apoptosis rates, demonstrating tumor‐suppressive effects in vivo.^228^

*YTHDF1* upregulation may inhibit antitumor immune responses, affecting apoptosis and programmed cell death. FTO inhibitors, including Compound 18097, augment the m^6^A modification in suppressor of cytokine signaling 1 (SOCS1) mRNA, which increases its stability and activates the P53 pathway, facilitating apoptosis.^229^ FTO inhibitor 44/ZLD115 upregulates RARA and downregulates MyC in leukemia cells, inducing apoptosis.^230^ IGF2BP2 regulates m^6^A modifications, affecting mitochondrial activity and gene expression in hematopoietic stem cells HSCs and, thereby, influencing cell survival and programmed death. Mitochondrial activity is closely linked to both energy metabolism and apoptosis.^231^ *IGF2BP1* knockdown induces cancer cell apoptosis; this effect highlights its role in programmed death. Cucurbitacin B (CuB), a chemogenetic small molecule, promotes apoptosis by blocking IGF2BP1's recognition of m^6^A‐modified mRNA, which affirms m^6^A's critical role in cancer cell survival and death regulation.^191^ High FTO expression inhibits apoptosis and promotes leukemia cell survival by affecting the PDGFRB/ERK signaling axis.^232^, ^233^ *IGF2BP2* knockdown reduces T‐ALL cell proliferation and increases apoptosis by modulating m^6^A modifications and interacting with NOTCH1.^234^

ADAR1 prevents the accumulation of Z‐RNA, inhibiting ZBP1 activation and RIPK3‐mediated necroptosis and helping cancer cells evade programmed cell death.^235^

Autophagy is a cellular process that involves the degradation and recycling of intracellular components to maintain homeostasis (Figure 4B). Under specific conditions, autophagy can result in cell death. m^6^A modifications can affect cell viability by altering the expression of genes associated with autophagy.^236^ The m^6^A methyltransferase METTL3 targets *DCP2*, influencing the regulation of the Pink1‐Parkin pathway, which, in turn, affects mitochondrial autophagy and damage in small‐cell lung cancer (SCLC) cells. This interaction contributes to chemotherapy resistance in SCLC patients.^237^ *METTL3* and *YTHDF1* facilitate the m^6^A modification of Rubicon mRNA, increasing transcript stability. Rubicon, in turn, inhibits the fusion process between autophagosomes and lysosomes, obstructing the clearance of LDs.^238^

Ferroptosis is an emerging form of programmed cell death characterized by the accumulation of intracellular iron and subsequent peroxidation of lipids^239^ (Figure 4C). FTO promotes OTU deubiquitinase, ubiquitin aldehyde binding 1 (OTUB1) expression by demethylating its m^6^A, inhibiting ferroptosis and increasing radioresistance.^240^ FTO acts as a tumor suppressor in PTC by downregulating the cystine/glutamate antiporter solute carrier family 7 member 11 (SLC7A11) via the ferroptosis pathway.^241^ METTL3 engages the m^6^A reader YTHDF1 to boost m^6^A modification and the translation of SLC7A11, facilitating LUAD cell proliferation and suppressing ferroptosis.^242^ The METTL3/IGF2BP1/m^6^A axis stabilizes SLC7A11 mRNA, upregulating its expression by preventing deadenylation. This upregulation facilitates hepatoblastoma (HB) cell proliferation and mitigates ferroptosis in vitro and in vivo.^243^ The upregulation of m^6^A–SLC7A11 in glioblastoma confers resistance to ferroptosis, suggesting that targeting the m^6^A–SLC7A11 axis could, instead, promote cancer cell ferroptosis.^244^

Hypoxia‐induced lncRNA–CBSLR interacts with YTHDF2, forming a signaling axis that reduces cystathionine beta‐synthase (CBS) mRNA stability. This affects the methylation and degradation of ACSL4 protein, lowering proferroptotic phosphatidylethanolamine levels and leading to ferroptosis resistance.^245^ CD8 T cells stimulate *ACSL4* expression via IFNγ, altering tumor cell lipid profiles and promoting the incorporation of arachidonic acid (AA) into phospholipids, inducing ferroptosis. Low‐dose AA further promotes this mechanism and boosts immune checkpoint blockade (ICB)‐induced antitumor immunity. Targeting the ACSL4 pathway could be an anticancer strategy.^246^ The inhibition of protein arginine methyltransferase 3 (PRMT3) leads to the upregulation of *METTL14*, which increases m^6^A–YTHDF2‐dependent methylation. This process diminishes the stability of glutathione peroxidase 4 (GPX4) mRNA, heightens lipid peroxidation, and accelerates ferroptosis in endometrial cancer.^247^ YTHDF1 suppresses CD8+ T cell‐induced ferroptosis in PCa, affecting programmed cell death. *YTHDF1* upregulation increases PD‐L1 expression, diminishing T cell cytotoxicity and ferroptosis; this effect affirms *YTHDF1*’s critical role in tumor immune evasion.^248^

### 5.2 Tumor immune microenvironment

**FIGURE 5:**
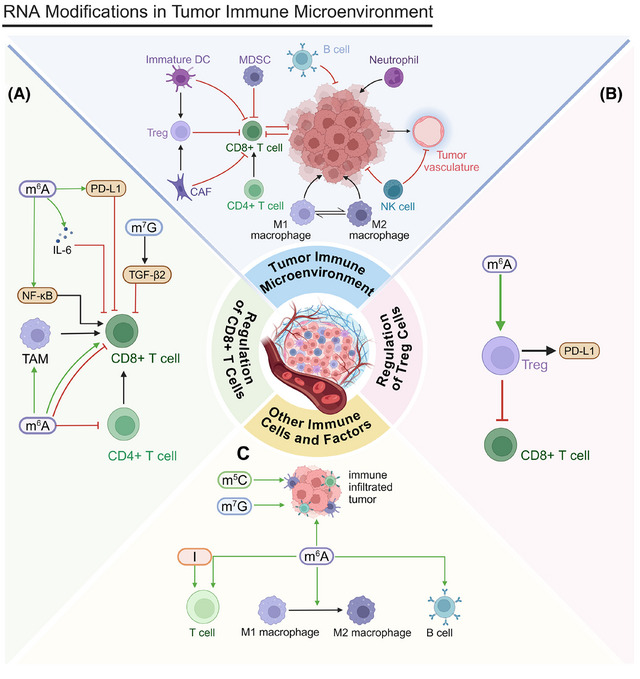
Tumor immune microenvironment. (A) Regulation of CD8+ T cells. METTL1 upregulation induces TGF‐β2 translation, inhibiting CD8+ T cell activity. YTHDF2 deficiency enhances TAM antigen presentation, promoting CD8+ T cell activation. High YTHDF2 expression reduces immune infiltration. ALKBH5 inhibits NF‐κB pathway, promoting CD8+ T cell infiltration. YTHDF1 induces IL‐6 secretion, inhibiting CD8+ T cell cytotoxicity. YTHDF1 depletion restores CD8+ T cell infiltration. IGF2BP1 increases PD‐L1 stability, reducing CD8+ T cell function. (B) Regulation of Treg cells. Treg cells inhibit effector T cell activity. In glycolytic tumors, Treg cells absorb lactate, promoting NFAT1 translocation and PD‐1 expression. YTHDF2 deficiency increases Treg cell apoptosis and impairs their suppressive function. (C) Regulation of other immune cells and factors. ALKBH5 overexpression inhibits RIG‐I‐mediated IFNα secretion. METTL14 regulates mRNA decay of negative immune regulators. TIMs’ immunosuppressive ability is regulated by METTL3. YTHDF3 promotes M1 macrophage polarization. IGF2BP1 is associated with immune checkpoint expression and TMB. m^6^A/m^5^C modifications influence immune cell infiltration and function. m^7^G modification affects tumor cell response to death signals. ADAR1 isoforms have dual roles in immune response.

The regulation of the tumor immune microenvironment is another key factor in cancer programmed cell death. CD8+ T cells serve as key effector cells that can directly eliminate tumor cells, and their infiltration levels within the TME are strongly linked to prognoses (Figure 5A). Boosting the infiltration and activity of CD8+ T cells is a primary objective of numerous immunotherapy approaches.

After RFA in HCC, *METTL1* upregulation induces *TGF‐β2* translation, creating an immunosuppressive environment and inhibiting CD8+ T cell activity. Fewer CD8+ T cells decrease tumor cells’ sensitivity to programmed cell death. *METTL1* influences tumor cell survival and programmed cell death by regulating the immune microenvironment.^249^ Within the TME, a deficiency in YTHDF2 augments the antigen‐presenting capacity of tumor‐associated macrophages (TAMs), facilitating the activation and proliferation of CD8+ T cells. Activated CD8+ T cells effectively recognize and kill tumor cells, promoting programmed cell death. Therefore, YTHDF2 affects tumor cell survival and death by regulating TAM function.^250^ Elevated *YTHDF2* expression in SCLC is associated with decreased immune infiltration, manifesting as reductions in cluster of differentiation 4 (CD4)+ and cluster of differentiation 8 (CD8)+ T cells, affecting immune‐related treatments and resulting in poor prognoses.^251^ ALKBH5 promotes CD8+ T cell infiltration in the CRC microenvironment by inhibiting the NF‐κB pathway and reducing C‐C motif chemokine ligand 5 (CCL5) expression. Increased CD8+ T cell infiltration may promote tumor cell programmed cell death.^252^

circCCAR1 induces CD8+ T cell dysfunction by stabilizing programmed cell death protein 1 (PD‐1), potentially reducing tumor cells’ sensitivity to programmed cell death. IGF2BP3 increases circCCAR1 stability, indirectly affecting tumor cell survival and death.^253^ YTHDF1 inhibits CD8+ T cell cytotoxicity by inducing IL‐6 secretion, allowing tumor cells to evade immune surveillance and inhibiting programmed cell death. This immunosuppressive environment facilitates tumor cells’ survival and proliferation.^254^ The reduction of YTHDF1 impedes tumor growth by reinstating CD8+ T cell infiltration, suggesting its significant role in programmed cell death.^255^ IGF2BP1 affects programmed cell death by reducing CD8+ T cell‐mediated tumor cytotoxicity. The upregulation of *IGF2BP1* increases PD‐L1 stability, inhibiting CD8+ T cell function and reducing apoptosis in CRC.^256^ Retinoic acid‐inducible gene I (RIG‐I) (encoded by DDX58 mRNA) influences immune cell activity through the interferon alpha (IFNα) signaling pathway. The upregulation of *ALKBH5* inhibits this mechanism, possibly enabling tumor cells to evade programmed cell death and, thereby, fostering tumor growth.^257^

Conversely, regulatory T (Treg) cells help tumor cells evade immune attack by inhibiting effector T cell activity (Figure 5B). Targeting Treg cells can improve antitumor immune responses.^258^ In tumors with high glycolytic activity, Treg cells actively uptake lactate through monocarboxylate transporter 1 (MCT1), which facilitates the translocation of nuclear factor of activated T‐cells 1 (NFAT1) to the nucleus and increases PD‐1 expression. Effector T cells, however, have suppressed PD‐1 expression. Lactate in the high‐glycolytic TME significantly affects cancer programmed cell death and immunotherapy efficacy by affecting Treg cells’ PD‐1 expression.^259^ YTHDF2 deficiency increases apoptosis and impairs the suppressive function of Treg cells in the TME. Reduced Treg cells may increase tumor cells’ sensitivity to programmed cell death as Treg cells typically protect tumor cells by inhibiting effector T cell activity.^260^

Beyond CD8+ T cells and Treg cells, numerous other critical immune cells and factors populate the tumor immune microenvironment (Figure 5C). Specifically, in head and neck squamous cell carcinoma (HNSCC), the overexpression of *ALKBH5* suppresses RIG‐I‐mediated IFNα secretion via the IκB kinase epsilon/TBK1/interferon regulatory factor 3 (IRF3) signaling pathway, which is closely related to tumor cell programmed cell death. In addition, the overexpression of *ALKBH5* diminishes the population of tumor‐infiltrating lymphocytes, which could reduce immune surveillance and programmed cell death.^257^ METTL14‐mediated m^6^A modification may influence tumor cell programmed cell death by regulating the mRNA decay of negative immune regulators. By promoting positive selection, METTL14 may increase B cells’ resistance to immune attacks.^261^

The immunosuppressive function of tumor‐infiltrating myeloid cells (TIMs) influences both tumor cell survival and the process of programmed cell death, primarily through METTL3 regulation. The presence of TIMs may inhibit T cell activity, affecting tumor cell programmed cell death.^262^ YTHDF3 may influence programmed cell death mechanisms by promoting M1 macrophage polarization, increasing the immune response against tumor cells, and increasing apoptosis rates. Conversely, M2 macrophage polarization may lead to immunosuppression and reduced tumor cell apoptosis.^263^ IGF2BP1 is linked to immune checkpoint expression and tumor mutational burden (TMB), potentially affecting programmed cell death mechanisms through immune pathways and altering cancer cell survival and death rates.^264^

m^6^A/m^5^C modifications can influence tumor cell sensitivity to programmed cell death by affecting the immune microenvironment. Early‐stage LUAD with high m^6^A/m^5^C expression presents with elevated immune checkpoint gene and immune cell expression, potentially enabling tumor cells to evade immune surveillance and reducing their sensitivity to programmed cell death.^265^ m^5^C modification regulates the immune microenvironment by modulating immune cell infiltration and function, which can alter cancer cells’ sensitivity to programmed cell death (e.g., immune‐mediated apoptosis), further aiding their survival.^266^, ^267^ m^7^G modification can affect tumor cells’ response to programmed death signals. Cancer patients with a high m^7^G risk also exhibit higher TMB and immunosuppressive states.^268^, ^269^, ^270^ ADAR1's two isoforms (p150 and p110) have dual roles in the immune response, indicating their potential regulation of cancer cell programmed death. The p150 isoform may increase immune surveillance in the TME by promoting T cell infiltration, facilitating tumor cell clearance. Conversely, silencing the constitutive p110 isoform reduces T cell chemotaxis, potentially allowing cancer cells to evade immune‐mediated programmed death and fostering their survival.^271^

### 5.3 Signaling pathway regulation

The regulation of signaling pathways is essential to orchestrate programmed cell death. RNA modifications affect tumor cell survival and death through various pathways, including the PD‐L1/PD‐1 and mTOR/AKT pathways. The PD‐L1/PD‐1 pathway is significant in immune evasion and tumor survival^272^; meanwhile, the mTOR/AKT pathway contributes crucially to governing cell growth and metabolic regulation.^273^

Seven in absentia homolog 2 (Siah2), a RING E3 ubiquitin ligase, plays a vital role in tumorigenesis and cancer progression. In cholangiocarcinoma (CCA), METTL14 increases m^6^A modifications in the 3′UTR region of Siah2 mRNA, leading to its degradation via a YTHDF2‐dependent mechanism. This degradation of Siah2 is crucial to sustaining PD‐L1 expression on tumor cells, which, in turn, inhibits T cell proliferation and cytotoxicity, modulating programmed cell death in CCA.^274^

METTL3 posttranscriptionally augments PD‐L1 expression in an IGF2BP3‐dependent fashion, stabilizing PD‐L1 mRNA. By inhibiting either METTL3 or IGF2BP3, antitumor immunity can be improved as this modification affects PD‐L1‐mediated T cell activation, exhaustion, and infiltration in vitro and in vivo.^20^, ^275^, ^276^, ^277^ METTL3 is essential for BCa cells to evade CD8+ T cell cytotoxicity as it controls PD‐L1 expression. In addition, JNK signaling facilitates tumor immune evasion through a METTL3‐dependent pathway.^278^ In HCC, LPS induces the upregulation of *METTL14*, which, in turn, increases the m^6^A methylation of MIR155HG. MIR155HG acts as a competing endogenous RNA, regulating PD‐L1 expression via the miR‐223/signal transducer and activator of transcription 1 (STAT1) axis, promoting immune evasion in HCC.^279^ In HCC, ALKBH5 increases the expression of *MAP3K8*, which then activates the JNK and ERK signaling pathways, regulating IL‐8 expression and promoting PD‐L1+ macrophage recruitment.^216^ METTL1 promotes PMN–MDSC accumulation via m^7^G, inhibiting tumor‐specific T cell activity and reducing ICC tumor cell sensitivity to PD‐1 therapy. PMN–MDSCs secrete inhibitory cytokines and express suppressive molecules, decreasing T cell function and affecting tumor cell survival and death.^280^ ALKBH5 affects T cell activity by regulating PD‐L1 expression. PD‐L1 upregulation typically inhibits T cell cytotoxicity, increasing tumor cell survival.^281^

Hypoxic conditions significantly elevate *YTHDF2* expression, which activates the mTOR/AKT signaling pathway in LUSC, increasing its antiapoptotic capabilities and reducing programmed cell death. The mTOR/AKT pathway facilitates cell proliferation and suppresses apoptosis via multiple mechanisms.^193^ mTORC1, a crucial element of the mTOR signaling pathway, governs cellular growth and metabolism, especially in response to nutrient availability and growth factor cues. Postchemotherapy, the upregulation of L‐amino acid transporter 2 increases amino acid uptake, activating mTORC1. This activation is associated with c‐Myc‐mediated cluster of differentiation 47 (*CD47*) transcription, where *CD47* upregulation may inhibit macrophage phagocytosis of tumor cells, inhibiting programmed cell death.^282^ The inhibition of *YTHDF1* results in the overexpression of the interferon‐gamma (IFN‐γ) receptor and activation of the JAK/STAT1 signaling pathways, which increase tumor cells’ sensitivity to the immune response.^283^ In HCC, m^6^A‐modified circMDK is upregulated, increasing cell proliferation, migration, and invasion. This is achieved by sponging miR‐346 and miR‐874‐3p and upregulating autophagy‐related 16 like 1 (ATG16L1), which activates the PI3K/AKT/mTOR signaling pathway.^284^

In conclusion, RNA modifications regulate various mechanisms of programmed cell death in cancer. A comprehensive exploration of the specific roles and mechanisms of RNA modifications can improve our understanding of tumor cell survival and apoptosis, providing new perspectives for developing therapeutic strategies.

## 6 RNA MODIFICATIONS IN CANCER THERAPY

The application of RNA modifications to cancer therapies is another idea that has garnered increasing attention. These modifications affect tumor cell responses through various mechanisms. In the following sections, we investigate the roles of RNA modifications in conventional therapies, immunotherapies, molecular targeted therapies, and metabolism‐related treatments.

### 6.1 Conventional therapies

Traditional cancer treatments include chemotherapy and radiotherapy. However, tumor cells often develop resistance to these therapies through various mechanisms. RNA modifications are essential to modulate tumors’ resistance to both chemotherapy and radiotherapy. By affecting drug metabolism, DNA repair, and apoptosis, RNA modifications can significantly alter tumor cells’ treatment sensitivity.

**FIGURE 6:**
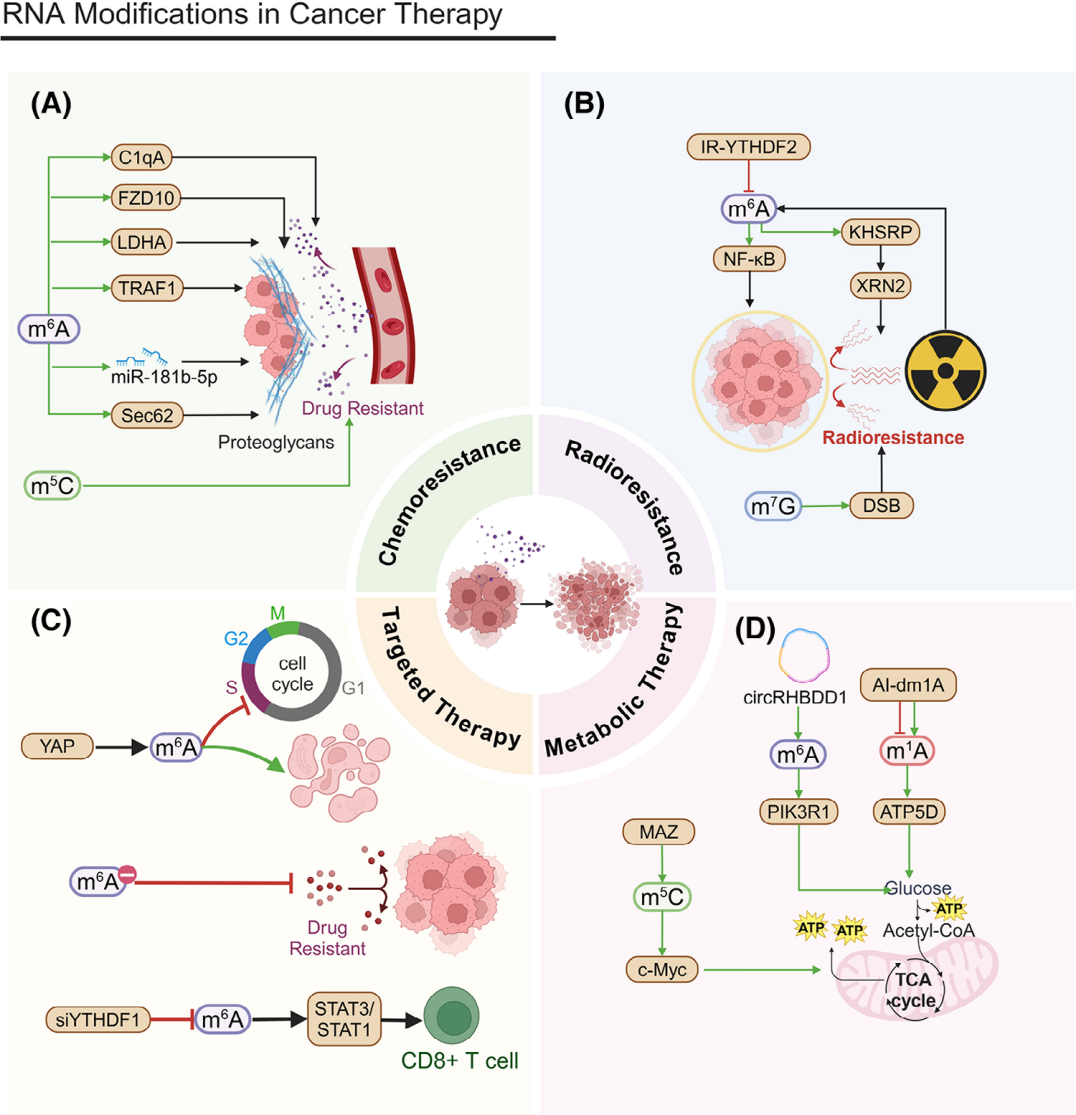
RNA modifications in cancer therapy. (A) Chemoresistance. METTL3/LDHA axis enhances 5‐FU resistance in CRC. METTL3‐dependent m^6^A methylation upregulates miR‐181b‐5p in CAFs, reducing 5‐FU sensitivity. METTL3‐mediated m^6^A modification upregulates Sec62, promoting CRC stemness and chemoresistance. METTL3 and YTHDF2 regulate C1qA methylation, affecting rituximab resistance in DLBCL. METTL3 enhances lenvatinib resistance in liver cancer via FZD10 m^6^A modification. METTL14‐dependent m^6^A modification stabilizes TRAF1, linked to sunitinib resistance. METTL16 correlates with PDAC sensitivity to PARP inhibitors. NSUN2 overexpression is associated with poor prognosis and reduced sensitivity to chemotherapeutic drugs. (B) Radioresistance. YTHDF2 inhibition enhances antitumor immunity and overcomes radioresistance via the IR–YTHDF2–NF‐κB circuit. Carbon ion radiotherapy increases METTL3 and m^6^A levels in NSCLC cells, suggesting new strategies. Bone‐specific m^6^A‐modified eRNA prevents XRN2 degradation, contributing to radiotherapy resistance in mPCa. METTL1 mediates m^7^G tRNA modifications, enhancing DSB repair and radiotherapy resistance in HCC. (C) Targeted therapy. In DLBCL, inactivating Hippo–YAP pathway via CHST11 and KIAA1429/YTHDF2 inhibits cell proliferation and induces apoptosis. METTL3 inhibitors are potential treatments for myeloid leukemia. METTL7B increases EGFR‐TKI resistance in LUAD cells, but knockdown resensitizes cells. M.RGD@Cr‐CTS–siYTHDF1 nanoparticles target TAMs, delivering siYTHDF1 and promoting CD8+ T cell infiltration. (D) Metabolic therapy. m^1^A regulates cancer cell glycolysis. AI‐dm1A tool modulates ATP5D expression and glycolysis. circRHBDD1 recruits YTHDF1 to PIK3R1 mRNA, enhances glycolysis and limiting anti‐PD‐1 therapy efficacy. NOP2 promotes HCC progression via aerobic glycolysis; NOP2 knockout combined with sorafenib increases sensitivity.

Chemoresistance presents a significant obstacle in cancer treatment (Figure 6A). RNA modifications affect tumor cells’ tolerance of chemotherapeutic agents by modulating the expression of drug‐metabolizing enzymes and drug efflux transporters.^285^, ^286^

Chemoresistance to 5‐fluorouracil (5‐FU) significantly contributes to poor prognoses in CRC patients. Resistant cells bolster 5‐FU resistance through the METTL3/lactate dehydrogenase A (LDHA) pathway. Inhibiting *METTL3* can restore chemosensitivity in CRC cells.^287^ In CRC, METTL3‐dependent m^6^A methylation upregulates miR‐181b‐5p in Cancer‐Associated Fibroblasts (CAFs), reducing 5‐FU sensitivity.^288^ METTL3‐mediated m^6^A modification also upregulates *Sec62*, promoting CRC stemness and chemoresistance through β‐catenin and Wnt signaling.^289^ Complement C1q subcomponent subunit A (C1qA) modulates rituximab resistance in DLBCL cells. The methylation of C1qA is regulated by METTL3 and YTHDF2, and knocking out these factors decreases rituximab resistance.^290^ METTL3 also increases lenvatinib resistance in liver cancer by regulating frizzled class receptor 10 (FZD10) m^6^A modification, activating the β‐catenin and YAP1 pathways.^219^ TNF receptor‐associated factor 1 (TRAF1) expression is linked to sunitinib resistance, and METTL14‐dependent m^6^A modification stabilizes TRAF1. Targeting METTL14 and TRAF1 may present new intervention strategies.^291^ *METTL16* expression correlates with PDAC cell sensitivity to Poly(ADP‐ribose) Polymerase (PARP) inhibitors, especially when combined with gemcitabine.^292^ *NSUN2* overexpression correlates with a poor prognosis in prostate cancer and decreased sensitivity to chemotherapeutic agents, such as docetaxel and cisplatin, underscoring the significance of the m^5^C modification to the chemotherapeutic response.^226^ RNA modification might alter treatment outcomes by regulating drug metabolism‐related genes or affecting immune infiltration in the TME.^293^

Elevated *METTL1* expression in tumor cells increases their sensitivity to specific chemotherapeutic drugs that target chromatin histone methylation, as well as the ERK–MAPK and WNT signaling pathways, suggesting METTL1 as a potential biomarker for drug sensitivity. Elevated *METTL1* expression is linked to effective responses to anti‐PD‐L1 therapy, highlighting its potential to guide immunotherapy.^209^ Beyond common RNA modifications, such as m^6^A and m^5^C, ncRNA itself is a crucial regulatory mechanism, playing key roles in various diseases, including cancer.^294^ Research on ncRNA combined with drug therapy represents a development of traditional cancer treatments.^295^, ^296^

As a novel approach to combat chemotherapy‐resistant tumors, an increasing number of clinical studies are being conducted utilizing RNA modification and RNA sequencing, offering promising prospects for cancer treatment (Table 1). Current RNA‐focused clinical research primarily targets two areas: Due to the minimally invasive nature and lower risk of RNA detection, which only requires blood or bodily fluids for extraction, RNA is predominantly studied as a biomarker for precancerous conditions and postoperative monitoring. Given the complexity of RNA regulatory mechanisms, inherent medical risks, and ethical considerations, direct RNA‐based therapeutic approaches remain limited. At present, RNA therapies are primarily employed in combination with traditional chemotherapeutic agents or monoclonal antibodies as RNA vaccines. In addition, most research is concentrated in Phase I/II clinical trials to assess the safety of RNA therapies. Much research remains before RNA therapies become a standard clinical treatment modality.

**TABLE 1:** Clinical trials of RNA therapies combined with chemotherapy.

Study identifier	Phase	Disease condition	Intervention	Status
NCT03206047	I/II	Platinum‐resistant epithelial ovarian, fallopian tube, or primary peritoneal carcinoma	Atezolizumab ± guadecitabine ± CDX ‐1401 vaccine + DNMT	Recruiting
NCT02901899	II	Recurrent platinum‐resistant ovarian cancer	Guadecitabine + pembrolizumab + DNMT	Completed
NCT03480152	I/II	Metastatic melanoma, Gastrointestinal and genitourinary cancers	Personalized mRNA vaccine	Completed
NCT04847050	II	Solid tumor malignancy, hematologic malignancy, leukemia, lymphoma, multiple myeloma	mRNA‐1273 vaccine, mRNA‐1273 vaccine booster	Completed
NCT06575452	Not applicable	Glioma	RNA diagnostic test: blood, urine, and tumor tissue samples; plasma samples	Not yet recruiting
NCT04724239	II	MSS/pMMR advanced colorectal cancer	Sintilimab, chidamide (histone deacetylase inhibitor; HDACi), bevacizumab	Completed
NCT05508399	Observational	Locally advanced gastric adenocarcinoma, G/GEJ adenocarcinoma	DNA panel and RNA sequencing	Recruiting
NCT03253107	Observational	Gastric cancer	mRNA and miRNA expression analysis, qRT‐PCR validation	Recruiting
NCT02190227	II	Invasive breast cancer	Tumor RNA disruption assay biopsy	Completed
NCT04683315	II	Pancreatic cancer	RNA expression profiling for PurIST subtyping; Drug: mFOLFIRINOX, gemcitabine/nab‐paclitaxel; Radiation: chemoradiation	Recruiting
NCT05916755	Observational	Triple‐negative breast cancer	Neoadjuvant chemotherapy (NACT) ± immune checkpoint inhibitor (ICI), multiomics analysis	Recruiting
NCT05854030	Observational	Lung neoplasm, squamous cell carcinoma	Serum exosomal miRNA analysis	Recruiting
NCT03997747	I	Recurrent glioblastoma	pp65 RNA‐loaded lipid particles (pp65 RNA‐LPs, DP1); RNA‐loaded lipid particles (RNA‐LPs, DP2)	Recruiting
NCT01663285	II	Urothelial cancer, bladder cancer	Neoadjuvant cisplatin and gemcitabine; exploratory integrative tumor sequencing	Terminated
NCT03410693	II/III	Carcinoma, transitional cell	Drug: rogaratinib (BAY1163877); chemotherapy; RNA in situ hybridization (RNA‐ISH) for FGFR testing	Completed
NCT04367025	II	Gastric cancer	Drugs: camrelizumab, oxaliplatin, S1; single‐cell RNA sequencing for T cell expression analysis	Ongoing
NCT01374672	Observational	Localized osteosarcoma, metastatic osteosarcoma, osteoblastic osteosarcoma, recurrent osteosarcoma	Laboratory biomarker analysis (DNA and RNA methylation and transcription changes)	Completed
NCT03127111	Observational	Stage III colorectal cancer	Gene mutation analysis; gene methylation analysis; gene expression analysis; whole‐genome bisulfite sequencing; RNA‐sequencing (RNA‐seq); genome‐wide association study (GWAS)	Not yet recruiting
NCT04914026	Observational	Testicular germ cell cancer, seminoma, nonseminoma testicular cancer, stage I testicular cancer	Biomarker analysis (miR371)	Recruiting
NCT05839834	Observational	Neoplasms, cancer	Diagnostic test: blood test (RNA‐based model)	Recruiting
NCT05660408	I	Recurrent pulmonary osteosarcoma, recurrent high‐grade glioma	pp65 RNA LP (DP1); pp65/tumor mRNA RNA‐LP (DP2)	Recruiting
NCT06604130	Not applicable	Prostate cancer	Diagnostic test: plasma exosome RNA combination for prostate cancer screening	Enrolling by invitation
NCT00672542	I	Metastatic melanoma, absence of CNS metastases	Proteasome siRNA and tumor antigen RNA‐transfected dendritic cells	Completed
NCT02316457	I	Triple‐negative breast cancer	RNA‐based IVAC_W_bre1_uID; RNA‐based IVAC_W_bre1_uID/IVAC_M_uID	Completed
NCT00108264	I	Prostate cancer	Tumor RNA transfected dendritic cells	Completed
NCT04335890	I	Melanoma, uveal metastatic	Vaccination with IKKb matured dendritic cells loaded with autologous tumor‐RNA + RNA coding for defined antigens and driver mutations	Ongoing
NCT05202561	I	Advanced solid tumor	RNA tumor vaccine/RNA tumor vaccine + navuliumab	Ongoing
NCT05697224	Not applicable	Bladder cancer	Diagnostic test: urinary microRNA detection	Not yet recruiting
NCT04765410	Observational	Pancreatic adenocarcinoma	qRT‐PCR analysis of tissue microRNA profile via EUS‐FNA	Ongoing
NCT06432413	Observational	Colorectal cancer	Quantitative real‐time PCR for SNHG3 and LUNAR1 in serum	Completed
NCT05708209	Observational	Oral squamous cell carcinoma	Quantitative real‐time PCR for MALAT1 and miRNA‐124 in saliva	Completed
NCT03000764	Not applicable	Breast carcinoma, fibrosis	RNA expression analysis; skin biopsies; blood samples	Completed
NCT06459895	Observational	Bronchial asthma	Diagnostic test: Long noncoding MALAT1 gene expression assay	Completed
NCT03524430	Not applicable	Breast neoplasm female	Procedure: core needle biopsy for RNA disruption assay (RDA)	Recruiting
NCT05264974	I	Melanoma	Autologous total tumor mRNA‐loaded DOTAP liposome vaccine	Recruiting
NCT04573140	I/II	Adult glioblastoma, high‐grade glioma, WHO grade III or IV malignant glioma	RNA‐LP vaccines (autologous total tumor mRNA and pp65 full length LAMP mRNA‐loaded DOTAP liposome vaccine)	Recruiting

*Data source*: Clinical data obtained from https://clinicaltrials.gov.

**TABLE 2:** Impact of RNA modifications on cancer types: inhibition and promotion.

RNA modification type	Regulator	Cancer type	Inhibition/promotion	Data source	Citation
m^1^A	ALKBH1	CRC	Promotion	GenomicScape (177)	^81^
	TRMT6/61A	Bladder cancer	Promotion	Organization (5)	^328^
	TRMT6/DNMT3B/DNMT1/WTAP	GMA	Promotion	CGGA (693)	^329^
	YTHDC1	Glioma	Inhibition	TCGA (674)	^330^
				CGGA (657)	
	TRMT6/10C/61B	Glioma	Promotion	TCGA (674)	^330^
	ALKBH1/3			CGGA (657)	
	YTHDF1/2/3				
m^5^C	NSUN4	HCC	Promotion	TCGA (787)	^331^
	NSUN2	HCC	Promotion	Organization (55)	^332^
	NSUN2	GC	Promotion	TCGA (449)	^333^
	TRDMT1/NSUN6	CRC	Promotion	TCGA (547)	^334^
	/ALKBH1				
	NSUN6	PC	Inhibition	Organization (744)	^335^
	NSUN5	Glioma	Inhibition	GSEA (1001)	^336^
	NSUN3/NSUN4	LUSC	Promotion	TCGA (551)	^337^
m^6^A	METTL3	CC	Promotion	Organization (60)	^338^
	METTL14	TSCC	Inhibition	Organization (25)	^339^
	METTL14	BC	Promotion	Organization (332)	^340^
	METTL3	CRC	Inhibition	Organization (136)	^341^
	ALKBH5	HNSCC	Promotion	Organization (138)	^257^
	ALKBH5	NSCLC	Inhibition	Organization (60)	^342^
	YBX1	AML	Promotion	TCGA (1068)	^343^
	YTHDC1	PDAC	Inhibition	Organization (90)	^344^
	FTO	PTC	Inhibition	Organization (86)	^241^
m^7^G	METTL1/WDR4	HNSCC	Promotion	Organization (209)	^345^
				TCGA (546)	
				CCLE (1478)	
	METTL1/WDR4	ICC	Promotion	Organization (83)	^346^
	METTL1	BC	Promotion	Organization (174)	^105^
	METTL1	ESCC	Promotion	Organization (120)	^347^
	METTL1	GBM/AML	Promotion	Mouse (27)	^96^
				TCGA (33)	
	WBSCR22	PDAC	Inhibition	TCGA (103)	^102^
	WBSCR22	GBM	Promotion	GEPIA (162)	^103^
	WDR4	HCC	Promotion	TCGA (680)	^106^
	WDR4	LC		TCGA (571)	^348^
ψ	DKC1	CRC	Promotion	Organization (130)	^349^
	DKC1	CRC	Promotion	Organization (411)	^350^
	DKC1	HCC	Promotion	Organization (332)	^351^
	PUS10	LC	Inhibition	NJMU (5543)	^352^
				FLCCA (8881)	
	PUS7	Glioblastoma	Promotion	REMBRANDT (247)	^353^
				TCGA (160)	
				Gravendeel (167)	
	PUS7	OV	Promotion	TCGA (593)	^354^
I	ADAR1	GC	Promotion	Organization (76)	^355^
	ADAR1	PTC	Promotion	Organization (6)	^356^
	ADAR1	ESCC	Promotion	TCGA (89)	^357^
	ADAR1	Pancreatic cancer	Promotion	Organization (104)	^194^
	ADAR1/METTL3	Glioblastoma	Promotion	Organization (16)	^175^
	ADAR1	Prostate cancer	Promotion	Organization (28)	^358^
	ADAR1	Melanoma	Inhibition	Organization (36)	^359^
	ADAR1	Melanoma	Inhibition	TCGA (212)	^360^

Abbreviations: ADAR1, adenosine deaminase acting on RNA 1; ALKBH1, alkB homolog 1; ALKBH5, alkB homolog 5; AML, acute myeloid leukemia; BC, breast cancer; CC, cervical cancer; CRC, colorectal cancer; DKC1, dyskerin pseudouridine synthase 1; ESCC, esophageal squamous cell carcinoma; FTO, fat mass and obesity‐associated protein; GBM, glioblastoma; GC, gastric cancer; HB, hepatoblastoma; HCC, hepatocellular carcinoma; hnRNPA2B1, heterogeneous nuclear ribonucleoproteins A2/B1; HNSCC, head and neck squamous cell carcinoma; ICC, intrahepatic cholangiocarcinoma; IGF2BPs, insulin‐like growth factor 2 mRNA‐binding proteins; LC, lung cancer; LUAD, lung adenocarcinoma; METTL14, methyltransferase‐like 14; METTL3, methyltransferase‐like 3; METTL5, methyltransferase‐like 5; NSCLC, non‐small cell lung cancer; OSCC, oral squamous cell carcinoma; OV, ovarian cancer; PDAC, pancreatic ductal adenocarcinoma; PTC, papillary thyroid carcinoma; PUS10, pseudouridylate synthase 10; PUS7, pseudouridylate synthase 7; TNBC, triple negative breast cancer; TSCC, tongue squamous cell carcinoma; WDR4, WD repeat domain 4; WTAP, Wilms’ tumor 1 associated protein; YTHDC1, YTH domain‐containing protein 1; YTHDC2, YTH domain‐containing protein 2; YTHDFs, YTH domain‐containing family proteins.

Radiotherapy resistance affects cancer treatment outcomes (Figure 6B). RNA modifications regulate DNA re2pair and antioxidant genes, influencing the tumor response. MDSCs expand after radiotherapy, causing radioresistance and immunosuppression. *YTHDF2 inhibition*, via the ionizing radiation (IR)‐YTHDF2‐NF‐κB circuit, enhances antitumor immunity and overcomes radioresistance, making it a promising target for RT and RT/immunotherapy combinations.^297^ Carbon ion radiotherapy increases the METTL3 and m^6^A levels in NSCLC cells. Targeting m^6^A and METTL3 may offer new strategies by affecting cancer cell proliferation, migration, and survival.^221^ Bone‐specific m^6^A‐modified eRNA is essential for mediating radiotherapy resistance in metastatic prostate cancer (mPCa) by averting XRN2 degradation through KHSRP. Disrupting the MLXIPe/KHSRP/PSMD9 complex suppresses tumor growth and heightens radiotherapy sensitivity.^298^ METTL1 increases radiotherapy resistance in HCC by mediating m^7^G tRNA modifications and promoting Double‐Strand Break repair. High METTL1 expression correlates with poor radiotherapy outcomes in HCC patients.^299^

### 6.2 Immunotherapy

Immunotherapy represents a major advancement in cancer treatment. RNA modifications modulate the efficacy of checkpoint inhibitors, facilitating tumor immune evasion, and transforming cold tumors into hot tumors. In addition, these modifications affect the immune response within the TME by regulating inflammatory mediators and the infiltration of immune cells.

**FIGURE 7:**
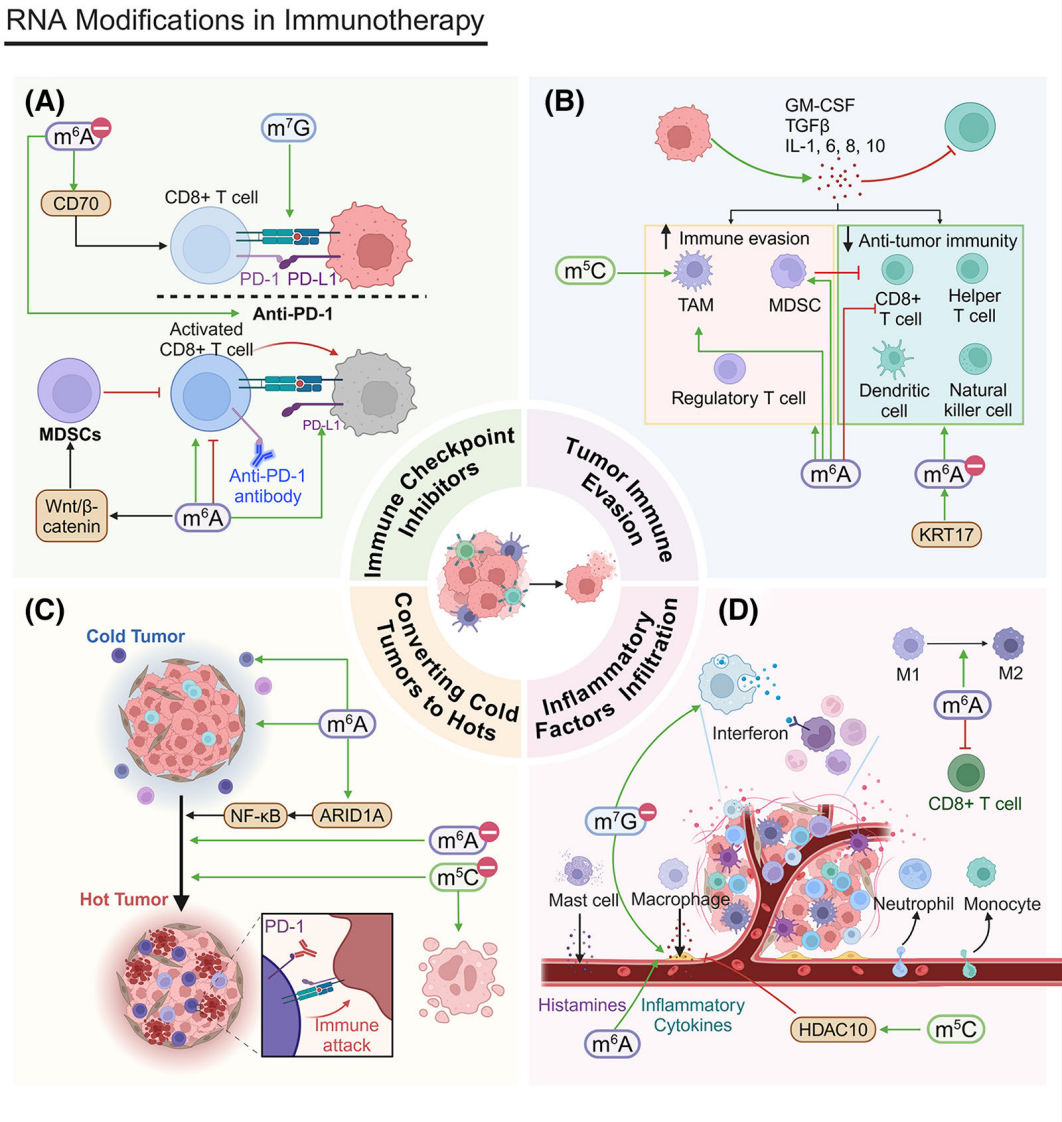
RNA modifications in immunotherapy. (A) Immune checkpoint inhibitors. m^6^A modifications play a crucial role in immune checkpoint inhibitors, with reduced METTL16 increasing PD‐L1 and decreasing PD‐1 positive T cells, thus reducing immunotherapy efficacy; ALKBH5 demethylates AXIN2 mRNA, enhancing Wnt/β‐catenin signaling and recruiting MDSCs, causing CRC immunosuppression; YTHDF1 depletion restores CD8+ T cell infiltration, enhancing PD‐1 blockade efficacy; IGF2BP1 regulates PD‐L1 expression, affecting antitumor immunity; low METTL3 expression stabilizes CD70 mRNA, promoting an immunosuppressive microenvironment, blocking CD70 can reverse anti‐PD‐1 resistance; METTL3 prevents YTHDF2‐mediated NLRC5 mRNA degradation, enhancing immune surveillance. Additionally, PRMT3‐mediated METTL14 methylation promotes EC progression and resistance, blocking PRMT3 enhances anti‐PD‐1 efficacy; the m^7^G risk score identifies CRC patients who may benefit from ICIs, optimizing immunotherapy strategies. (B) Tumor immune evasion: m^6^A modifications reduce CD8+ T cell infiltration, promoting immune evasion, METTL3 in TIMs enhances JAK1 translation and STAT3 phosphorylation, METTL3‐induced circMYO1C stabilizes PD‐L1, promoting PDAC immune evasion, silencing METTL3 reduces MDSC accumulation, maintaining CD4+ and CD8+ T cell activation, KRT17 promotes YTHDF2 degradation, enhancing T cell infiltration and CXCL10 expression. NSUN5 enhances TAM phagocytosis by regulating m^5^C‐modified RNA and inhibiting β‐catenin signaling. (C) Converting cold tumors to hot tumors. High‐risk HCC patients show increased immunosuppressive cell infiltration, with mfrlncRNA playing a crucial role, IMS2 in PCa shows a cold phenotype and poor prognosis, with HNRNPC as a biomarker, YTHDF1 deficiency limits MHC‐I and antigen lysosomal proteolysis, restoring tumor immune surveillance, YTHDF2 targets Treg cells, boosting antitumor immunity, deleting the glucose/NSUN2/TREX2 axis enhances apoptosis and CD8+ T cell infiltration, TA and FTO inhibitor nanoparticles enhance T cell infiltration and immune memory. (D) Inflammatory factors and immune cell infiltration. METTL1‐mediated m^7^G modification protects tRNA from stress‐induced cleavage, loss of m^7^G methylation activates the interferon pathway, knocking down METTL1 increases proinflammatory immune cell infiltration, IGF2BP3 promotes macrophage infiltration and M2 polarization by enhancing CCL5 and TGF‐β1 expression, NSUN6 promotes HDAC10 expression, inhibiting macrophage‐related chemokine transcription and M2 macrophage recruitment.

Immune checkpoint inhibitors (ICIs) boost antitumor immunity by relieving the immune system's suppression of tumor cells (Figure 7A). RNA modifications control the expression levels of checkpoint molecules, influencing the effectiveness of these inhibitors.

Many CRC patients respond poorly to ICI therapy. Reduced METTL16 and elevated PD‐L1 were observed in CRC tissues and cell lines. The overexpression of *METTL16* in CRC cells reduces the presence of PD‐1‐positive T cells. The METTL16/PD‐L1/PD‐1 axis is a promising therapeutic target.^300^ ALKBH5 demethylates axis inhibition protein 2 (AXIN2) mRNA, leading to its degradation and Wnt/β‐catenin overactivation, inducing Dickkopf‐related protein 1 (DKK1) and recruiting MDSCs and, therefore, driving CRC immunosuppression and tumorigenesis. Targeting ALKBH5 could sensitize CRC to immunotherapy.^301^ The depletion of YTHDF1 suppresses tumor growth by promoting CD8+ T cell infiltration, and this effect increases when combined with PD‐1 blockade. Clinically, high *YTHDF1* expression is linked to poor prognoses and suboptimal immunotherapy outcomes.^255^ IGF2BP1 influences antitumor immunity by modulating PD‐L1 expression in colon and liver cancers. Targeting IGF2BP1 could improve the effectiveness of PD‐1/PD‐L1 blockade therapies.^212^, ^256^ The reduced expression of METTL3 is associated with resistance to anti‐PD‐1 therapy. *METTL3* downregulation stabilizes CD70 mRNA, promoting an immunosuppressive microenvironment. Inhibiting CD70 using cusatuzumab can counteract M2 macrophage‐induced resistance to anti‐PD‐1 therapy, highlighting METTL3's role in regulating the immunotherapy response.^302^

In EC cells and CD8+ T cell coculture, elevated METTL3 expression suppresses the proliferation and migration of endometrial carcinoma (EC) cells while encouraging the proliferation of CD8+ T cells. Conversely, the depletion of METTL3 yields the opposite effect. METTL3 bolsters immune surveillance by hindering the YTHDF2‐mediated degradation of NLRC5 mRNA, indicating the METTL3/YTHDF2/NLRC5 axis as a potential target for EC immunotherapy.^303^ The arginine methylation of *METTL14* by PRMT3 facilitates the progression of EC and contributes to treatment resistance. Blocking PRMT3 sensitizes EC to immunotherapy and increases anti‐PD‐1 efficacy by accelerating ferritin deposition.^247^ The m^7^G risk score (MRS) can be used to identify CRC patients who may benefit from ICIs. To optimize treatment strategies, m^7^G‐related gene expression can be assessed.^304^

Tumor immune evasion refers to the strategies employed by tumor cells to escape attacks from the immune system (Figure 7B). RNA modifications influence immune evasion by regulating relevant gene expression.^200^ m^6^A modifications affect cancer immunotherapy by regulating immune cell functions. The aberrant expression of m^6^A methyltransferases or demethylases is linked to CD8+ T cell infiltration, as well as tumor immune evasion.^204^, ^305^ METTL3 mediates m^6^A modifications on Jak1 mRNA in TIMs, increasing JAK1 translation and STAT3 phosphorylation. Elevated METTL3 expression in TIMs is associated with a poor prognosis in colon cancer patients. Conversely, a deficiency of METTL3 in myeloid cells reduces tumor growth in mouse models.^262^ Targeting METTL3 could present new avenues for cancer immunotherapies. METTL3‐induced circMYO1C promotes PDAC proliferation and migration in vitro and increases immune evasion by stabilizing PD‐L1. Regulating m^6^A modifications may offer new strategies for PDAC immunotherapies as well.^306^ *METTL3* knockdown in CRC cells diminishes the accumulation of MDSCs and sustains the activation and proliferation of CD4+ and CD8+ T cells. Inhibiting METTL3 could counteract immunosuppression and bolster antitumor immunity.^205^ Keratin 17 KRT1 increases T cell infiltration in CRC by promoting YTHDF2 degradation via the ubiquitin–proteasome system. This increases CXC motif chemokine ligand 10 (CXCL10) expression, improving tumors’ response to immunotherapy and highlighting RNA modifications’ role in immune evasion.^307^ NSUN5 increases TAM phagocytosis in glioma by regulating m^5^C‐modified RNA and inhibiting β‐catenin. It interacts with and degrades CTNNB1 caRNA, linking its function to DNA methylation and emphasizing RNA modifications’ importance in the tumor immune microenvironment.^308^

Cold tumors lack immune cell infiltration and are often unresponsive to immunotherapy (Figure 7C). RNA modifications can convert cold tumors to hot tumors by upregulating immune cell recruitment and activation, increasing immunotherapy efficacy.^309^, ^310^

In HCC, m^6^A methylation and ferroptosis‐related lncRNA (mfrlncRNA) signatures have significant prognostic value, predicting both survival and risk stratification. High‐risk patients show increased immunosuppressive cell infiltration and reduced cytotoxic immune cell infiltration, suggesting mfrlncRNA's role in converting cold tumors to hot tumors.^305^ In PCa, m^6^A‐identified immune microenvironment subtype 2 (IMS2) shows a cold phenotype and a poor prognosis. HNRNPC serves as a biomarker for IMS2 and is also a promising therapeutic target to increase the efficacy of immunotherapy.^311^ NF‐κB, a key transcription factor, regulates apoptosis and survival signals. Regulating the ARID1A/NF‐κB pathway can improve the immunotherapy response.^312^

YTHDF1 deficiency inhibits lysosomal gene translation, limiting major histocompatibility complex class I (MHC‐I) and antigen lysosomal proteolysis and, thereby, restoring tumor immune surveillance. It also converts cold tumors to hot ones, increasing ICI efficacy.^313^ YTHDF2 targets Treg cells in the TME, boosting antitumor immunity without affecting peripheral immune homeostasis, reducing unnecessary inflammation, and improving immunotherapy outcomes.^260^ NSUN2, a glucose sensor, maintains TREX2 expression upon glucose activation, promoting tumor cell proliferation by inactivating cGAS/STING. Deleting the glucose/NSUN2/TREX2 axis inhibits tumorigenesis and increases apoptosis and CD8+ T cell infiltration, overcoming resistance to anti‐PD‐L1 therapy in cold tumors.^65^ The thermal ablation (TA) of HCC has high recurrence and metastasis rates. Xiao et al.^314^ created a nanoparticle platform that releases tumor‐associated antigens and FTO inhibitors to dendritic cells (DCs). In vivo, postintratumoral administration of these nanoparticles, they facilitate DC maturation, boost T cell infiltration, and establish immune memory. This synergizes with ICB therapy to suppress the growth of distant tumors and prevent metastasis.^314^

Inflammatory factors and the infiltration of immune cells are essential components of the TME (Figure 7D). RNA modifications modulate the expression of genes associated with these factors, affecting the immune response within the TME.^315^ The METTL1‐mediated m^7^G modification of tRNA protects them from stress‐induced cleavage. The loss of tRNA m^7^G methylation activates the interferon pathway, making tumor cells stress‐sensitive.^316^ Reducing *METTL1* expression in prostate cancer models results in increased proinflammatory immune cell infiltration and improves the response to immunotherapy, suggesting METTL1 as a potential target to improve chemotherapy sensitivity.^317^ IGF2BP3, an m^6^A reader, facilitates macrophage infiltration and M2 polarization by upregulating the expression of CCL5 and transforming growth factor beta 1 (TGF‐β1), which, in turn, inhibits CD8+ T cell activation and contributes to the progression of liver cancer. The combination of IGF2BP3 inhibition with anti‐CD47 therapy markedly suppresses liver cancer growth.^318^ In the BCa TME, NSUN6 mediates m^5^C methylation to promote histone deacetylase 10 (HDAC10) expression, inhibiting macrophage‐related chemokine transcription and M2 macrophage recruitment. This provides insights into epigenetic regulation in TME and potential immunotherapy strategies.^319^

### 6.3 Molecular targeted therapy

Molecular targeted therapy offers effective treatment with fewer side effects by targeting key molecules in tumor cells (Figure 6C). RNA modifications affect these therapies by regulating key molecules’ expression and function.

In DLBCL, Hippo–YAP pathway inactivation via carbohydrate sulfotransferase 11 (CHST11) and KIAA1429/YTHDF2 repression inhibits cell proliferation and tumor growth and induces cell cycle arrest and apoptosis.^320^ METTL3 inhibitors are potential treatments for myeloid leukemia, according to Yankova et al.^321^ Increased METTL7B in drug‐resistant LUAD cells induces EGFR‐TKI resistance, but *METTL7B* knockdown resensitizes cells to gefitinib and osimertinib.^322^ A photosensitive, dual‐targeting nanoparticle system (M.RGD@Cr‐CTS)–small interfering RNA‐targeting YTHDF1 (siYTHDF1) nanoparticles target TAMs to deliver the drug, altering the STAT3/STAT1 balance, reducing IL‐10, increasing IL‐12 and IFN‐γ, and promoting CD8+ T cell infiltration, highlighting the potential of RNA interference in cancer immunotherapy.^323^

### 6.4 Metabolism‐related therapies

Metabolic therapies target tumor cell metabolic pathways, offering a novel treatment approach (Figure 6D). RNA modifications are essential in the regulation of tumor metabolic reprogramming, as they affect the expression of metabolic enzymes and pathways. This, in turn, alters both energy metabolism and biosynthetic processes within the tumor.

m^1^A regulates cancer cell glycolysis.^227^ Xie et al.^324^ developed an abscisic acid‐induced reversible m^1^A demethylation tool (AI‐dm^1^A) to facilitate photoinduced m^1^A methylation or demethylation. AI‐dm^1^A specifically reduces m^1^A levels in ATP synthase subunit delta (ATP5D), promoting ATP5D expression and increasing glycolysis. This m^1^A editing tool offers a flexible approach to studying m^1^A's effects on tumors and holds therapeutic potential.^324^ circRHBDD1 increases glycolysis and recruits the m^6^A reader YTHDF1 to PI3K regulatory subunit 1 (PIK3R1) mRNA, limiting the efficacy of anti‐PD‐1 therapy. Targeting circRHBDD1 may improve anti‐PD‐1 therapy's effects in HCC patients.^325^ NOP2 promotes HCC progression via MAZ/NOP2/c‐Myc‐mediated aerobic glycolysis. *NOP2* knockdown combined with sorafenib increases sorafenib sensitivity, significantly inhibiting tumor growth.^152^

In summary, RNA modifications play a critical role in various cancer therapy mechanisms. By thoroughly investigating the specific functions and mechanisms of these modifications, we can better understand tumor cells’ responses to different treatments and develop novel therapeutic strategies.

## 7 CONCLUSION AND PROSPECTS

This review explored RNA modifications, such as m^6^A, m^5^C, m^7^G, and m^1^A, and their regulatory factors: writers, erasers, and readers. We also introduced the writers involved in Ψ and adenosine‐to‐inosine changes. These regulators participate in key biological processes, including metabolic reprogramming (e.g., glycolysis, glutaminolysis, and lipid metabolism), biosynthetic pathways (e.g., ribosome and nucleic acid synthesis), signaling pathway regulation (e.g., PI3K/AKT/mTOR), and cell cycle control, which all affect tumor proliferation. In addition, these RNA modifications influence metabolism and the microenvironment, providing energy and a foundation for tumor metastasis.

A critical mechanism of cancer metastasis is EMT, which increases cancer cell migration by converting epithelial cells to mesenchymal cells, promoting tumor metastasis. RNA modifications regulate EMT‐related signaling pathways, altering gene expression and affecting cancer proliferation, metastasis, and stemness.^326^ Furthermore, RNA modifications play a crucial role in programmed cell death, including apoptosis, autophagy, and ferroptosis. These modifications influence cancer cell survival strategies by regulating pathways such as PD‐L1/PD‐1 and mTOR/AKT, as well as the immune microenvironment (e.g., CD8+ T cell infiltration and Treg cells), which presents new possibilities for cancer immunotherapies.

RNA modifications show great potential usefulness in cancer therapy, especially in targeted and immunotherapies. We summarized their roles in traditional treatments, such as chemotherapy and radiotherapy resistance. RNA modifications also regulate immune cell development, differentiation, proliferation, infiltration, activation, and apoptosis, affecting tumor immunotherapy. They also mediate immune checkpoint expression, promoting tumors’ immune evasion. Developing ICIs to target these RNA modification regulators could improve immunotherapy outcomes.

The conversion of “cold” tumors to “hot” tumors is crucial in cancer treatment as it reactivates the immune system through ICIs and immune suppressive cells, increasing the antitumor response.^327^ RNA modifications play a significant role in this conversion, offering new strategies to overcome immune evasion and develop more effective cancer therapies.

Given that RNA modifications significantly affect cancer proliferation, metastasis, and programmed cell death, they present promising targets for cancer treatment.

RNA modifications play crucial roles at various stages of cancer, exerting broad and complex effects. RNA modifications can reduce the stability of tumor suppressor genes, overcoming their inhibitory effects and fostering cancer progression. Meanwhile, these modifications can increase the stability of oncogene transcripts, which further accelerates tumor development. However, certain RNA modifications may exhibit opposite effects in different cancer types or, even, within the same cancer type; this underscores the complexity and significance of RNA modifications in cancer (Table 2). Current research has only begun to uncover the basis of epigenetic regulation in cancer.

The diversity and complexity of RNA modifications indicate their immense potential as therapeutic targets. RNA modifications play key roles not only in cancer proliferation, metastasis, and programmed cell death but also, potentially, in immunotherapy. Developing small molecule inhibitors that target RNA modification sites and their associated enzymes could provide novel, more specific cancer treatments. Future research should focus on elucidating specific RNA modification mechanisms to develop more effective therapeutic strategies and improve patient survival and quality of life.

## AUTHOR CONTRIBUTIONS

Han Wu and Shi Chen conceived and designed the study. Han Wu wrote the manuscript. Han Wu constructed the figure. Dongxu Wang revised and edited the manuscript. Weiwei Liu revised the manuscript. Xiang Li, Yuyang Li, He Shi, and Yiwen Qing assisted in organizing the references. Bohe Shi, Yifei Tang, Zhuoyi Yan, and Hao Yang helped with spelling and formatting checks. All authors have read and approved the final manuscript.

## CONFLICT OF INTEREST STATEMENT

The authors declare that they have no conflict of interest.

### ETHICS STATEMENT

Not applicable.

## Data Availability

Not applicable.
